# Study on the inhibitory activity and mechanism of *Mentha haplocalyx* essential oil nanoemulsion against *Fusarium oxysporum* growth

**DOI:** 10.1038/s41598-024-67054-1

**Published:** 2024-07-11

**Authors:** Hongxin Liao, Jinrui Wen, Hongyan Nie, Cuiqiong Ling, Liyan Zhang, Furong Xu, Xian Dong

**Affiliations:** School of Chinese Materia Medica, Yunnan University of Chinese Medicine, Kunming, 650000 China

**Keywords:** Nanometer emulsion, Ultrasonic method, Antifungal mechanism, Metabolism pathways, Prevention of root rot, Biological techniques, Biotechnology, Chemical biology, Microbiology

## Abstract

*Mentha haplocalyx* essential oil (MEO) has demonstrated inhibitory effects on *Fusarium oxysporum*. Despite its environmentally friendly properties as a natural product, the limited water solubility of MEO restricts its practical application in the field. The use of nanoemulsion can improve bioavailability and provide an eco-friendly approach to prevent and control *Panax notoginseng* root rot. In this study, Tween 80 and anhydrous ethanol (at a mass ratio of 3) were selected as carriers, and the ultrasonic method was utilized to produce a nanoemulsion of MEO (MNEO) with an average particle size of 26.07 nm. Compared to MTEO (MEO dissolved in an aqueous solution of 2% DMSO and 0.1% Tween 80), MNEO exhibited superior inhibition against *F. oxysporum* in terms of spore germination and hyphal growth. Transcriptomics and metabolomics results revealed that after MNEO treatment, the expression levels of certain genes related to glycolysis/gluconeogenesis, starch and sucrose metabolism were significantly suppressed along with the accumulation of metabolites, leading to energy metabolism disorder and growth stagnation in *F. oxysporum*. In contrast, the inhibitory effect from MTEO treatment was less pronounced. Furthermore, MNEO also demonstrated inhibition on meiosis, ribosome function, and ribosome biogenesis in *F. oxysporum* growth process. These findings suggest that MNEO possesses enhanced stability and antifungal activity, which effectively hinders *F. oxysporum* through inducing energy metabolism disorder, meiotic stagnation, as well as ribosome dysfunction, thus indicating its potential for development as a green pesticide for prevention and control *P. notoginseng* root rot caused by *F.oxyosporum*.

## Introduction

*Panax notoginseng*, a valuable Chinese herbal medicine derived from roots, is well-known for its therapeutic effects on cardiovascular and cerebrovascular diseases. Saponins, the main active medicinal ingredients, are recognized as valuable components in medicinal products^1^. In recent years, there has been an increasing demand for *P. notoginseng.* However, suitable land resources for its cultivation have diminished due to the “continuous cropping obstacle” and other factors^2^*.* During the growth process of *P. notoginseng*, it is susceptible to various diseases, with root rot caused by *F. oxysporum*, *F. solani,* and *P. herbarum* being the most common and destructive soil-borne disease affecting its cultivation and contributing to the continuous cropping obstacle^3^. Typical symptoms include yellowing of the leaves, collapsing stems, root rot and softening, and reduced numbers of smaller root hairs^4^. Statistics indicate an annual loss of about 5–20% due to root rot disease of *P. notoginseng*; severe cases can reach 70–85%, significantly hindering industry development^5^.

Currently, chemical pesticides are primarily used to control root rot disease in *P. notoginseng* cultivation. However, fungicides applied through root irrigation can lead to heavy metal pollution and pesticide residues that seriously impact quality and pharmaceutical value^6^. Physical control and microbial control are more environmentally friendly options. However, physical control is mainly preventive, while microbial control has the disadvantages of a slow effect and being susceptible to environmental influences, which are insufficient for preventing and controlling root rot in *P. notoginseng*. Plant essential oils (EOs), as secondary metabolites of plants, have demonstrated potent inhibitory effects on pathogenic fungi such as *F. oxysporum* and *F. solani* responsible for root rot^2^. The complex chemical composition of plant EOs confers them with a broad spectrum of antifungal activity, posing challenges for pathogenic fungi in developing resistance. Furthermore, plant EOs can function as signaling molecules in the natural environment to safeguard plants from damage^7^. Additionally, plant EOs readily decompose in natural environments and exert minimal impact on the environment, even aiding in mitigating heavy metal pollution in agricultural land^8^. Consequently, they represent promising candidates for the development of novel eco-friendly antifungal pesticides^9^.

Mentha *haplocalyx*, a perennial aromatic herb of the *Lamiaceae* family, is widely utilized in pharmaceutical and food industries due to its broad-spectrum antifungal activity exhibited by its EO^10^. Numerous studies have demonstrated the significant antifungal effects of *M. haplocalyx* EO (MEO) against various agricultural fungi. For instance, MEO effectively inhibits the proliferation of *Botryotinia fuckeliana*, *Curvularia hawaiiensis*, and *F. oxysporum*, thereby preventing crop infection and decay^11,12^. Furthermore, MEO significantly suppresses the growth of *Rhizopus stolonifer*, thereby reducing strawberry and peach fruit rot caused by *R. stolonifer*^13^, as well as papaya anthracnose induced by *Colletotrichum gloesporioides* and *Colletotrichum brevisporu*^14^*.* The potential for MEO to replace traditional fungicides in controlling fungus-induced spoilage of agricultural products makes it a promising candidate for development into a green fungicide that reduces reliance on chemical pesticides.

Like most EOs, MEOs are lipophilic liquids that are typically insoluble in water; this limits their bioavailability^15^. Additionally, the volatile nature of MEO components may reduce their antifungal efficacy due to low persistence^16^. Consequently, researchers have incorporated EOs into suitable delivery systems to enhance water solubility and antimicrobial activity^17^. Among these systems, water-in-oil nanoemulsions outperform traditional emulsions not only in enhancing active ingredient stability but also in ensuring good water solubility to increase the antimicrobial activity of EOs^18^. Therefore, an increasing number of researchers are dedicated to developing nanoemulsions of EOs. Nanoemusilfied EOs such as rosemary, eucalyptus, basil, and copaiba EOs have been used as larvicides and antimycotic agents^19,20^.

Nanoemulsions, consisting of two immiscible phases (oil and water) with particle sizes ranging from 10 to 100 nm, demonstrate enhanced stability compared to traditional emulsions regarding polymerization, flocculation, and precipitation^21^. Research has shown that the smaller particle size and larger particle area of nanoemulsions result in increased contact area with pathogens, thereby improving their antifungal activity^22^. The stability and antifungal efficacy of nanoemulsions are dependent on the choice of surfactants and the preparation method used. For instance, Tween 80, a synthetic surfactant widely utilized in producing nanoemulsions containing EOs, enhances both solubility and antifungal activity^23^. Furthermore, high-quality nanoemulsion preparation requires the use of high-energy sources such as ultrasound or high pressure to generate strong disruptive forces during homogenization process, which play a crucial role in determining particle size, stability, and functionality^24^. While nanoemulsions can enhance the solubility and stability of MEO, it is important to consider the concentration, frequency, and dosage of MEO nanoemulsions (MNEO) for achieving optimal control effect.

The diverse compositions of plant EOs confers upon them a wide range of antifungal activities by inhibiting multiple targets within pathogens. Some progress has been achieved in investigating their antifungal mechanisms. It has been documented that plant EOs and their primary components gradually disrupt fungal pathogens by inducing mycelial morphological changes, vacuole fusion, shedding of cell wall fibrous layer, and destruction of organelles^25,26^. The degradation and dissolution of the nucleus and mitochondria lead to the decomposition of cytoplasmic contents, ultimately causing the death of pathogens^25^. However, most studies on the antifungal mechanisms of plant EOs are currently limited to the cellular level; therefore, further understanding is required regarding their modes of action at microscopic levels, such as fungal gene expression levels and metabolite accumulation. This will facilitate a deeper understanding of the pathways through which plant EOs affect pathogens and how pathogens respond to stimulation by plant EOs.

The present study utilized Tween 80 and anhydrous ethanol as surfactants for the preparation of MNEO through ultrasonication to enhance their bioavailability. Various parameters were employed to evaluate the stability of the nanoemulsion and its inhibitory effect on *F. oxysporum*. Transcriptomics and metabolomics analyses were performed to investigate the inhibitory mechanism of MNEO and MEO dissolved in an aqueous solution of 2% DMSO and 0.1% Tween 80 (MTEO) against *F. oxysporum*, as well as their common and specific antifungal mechanisms, in order to elucidate the superior inhibitory effects of MNEO against *F. oxysporum*. In vivo experiments on *P. notoginseng* seedlings and rhizomes were also conducted to validate the practical effectiveness of MNEO in suppressing the causal agent of root rot in *P. notoginseng*.

## Results

### Stability of MNEO

**Figure 1:**
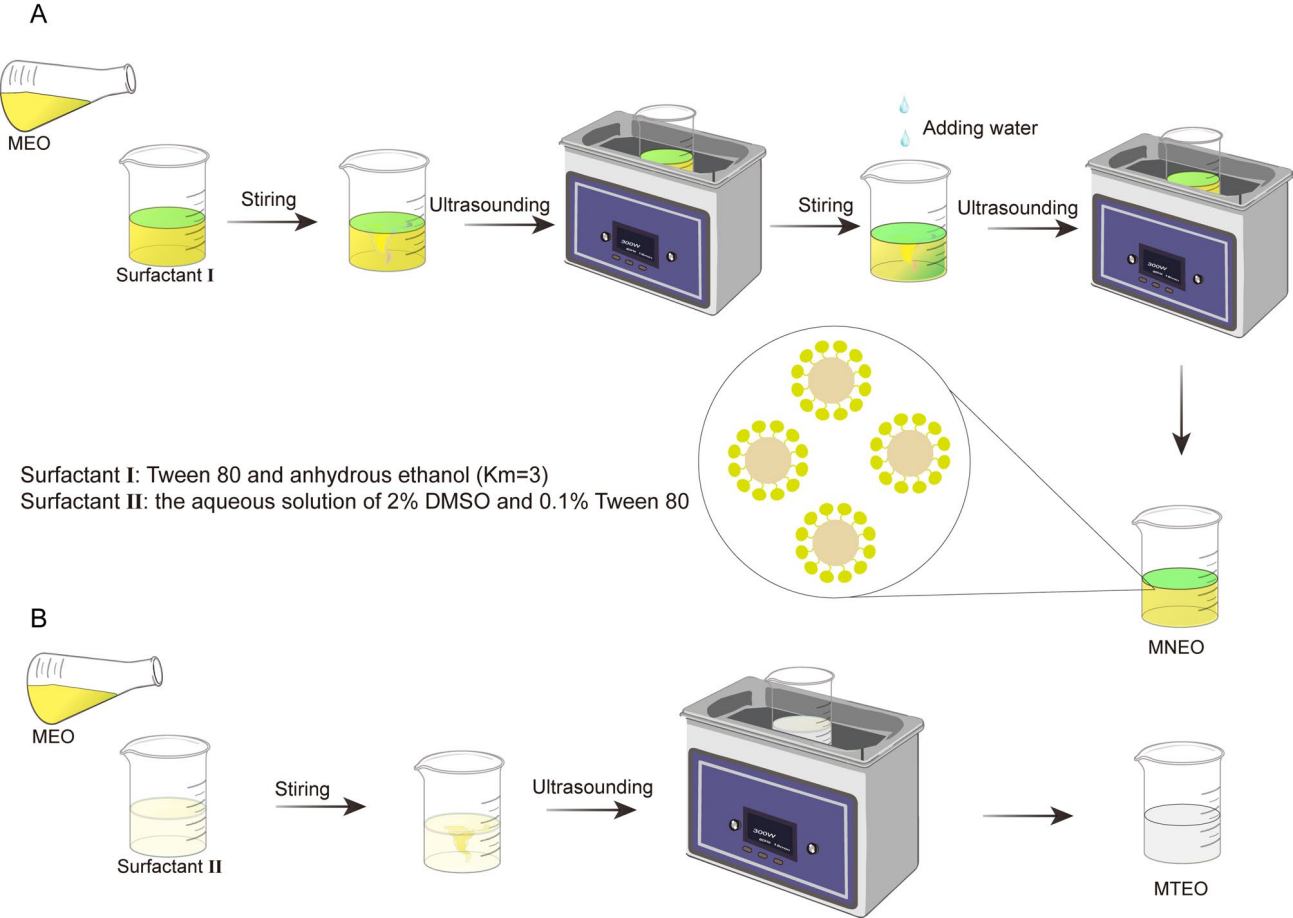
Flow chart of the production of emulsions. (**A**) The production process of MNEO. (**B**) The production process of MTEO.

**Figure 2:**
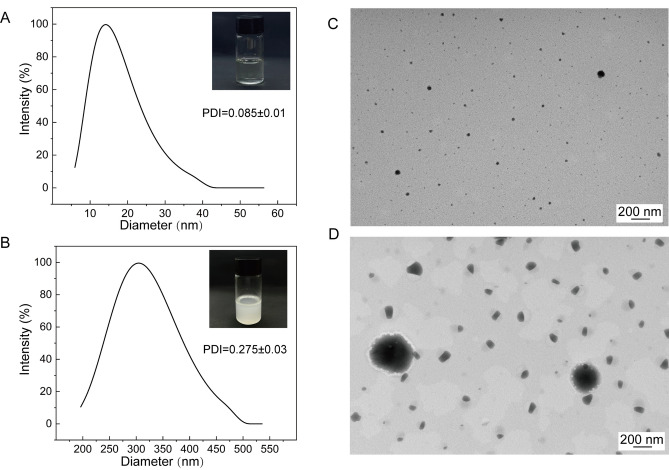
Particle size and transmission electron microscopy (TEM) images of MNEO and MTEO at 25 mg/mL. (**A**) The particle size distribution of MNEO was generated by particle size and its corresponding strength. (**B**) The particle size distribution of MTEO. (**C**) In the TEM image of MNEO, the acceleration voltage was 100 keV, and the morphology of MNEO was observed at a rate of 100,000 X. (**D**) In the TEM image of MTEO, the acceleration voltage was 100 keV, and the morphology of MNEO was observed at a rate of 50,000 X.

We prepared MNEO and MTEO according to the graphical method to prepare for subsequent experiments (Fig. 1). The particle size of the MNEO was measured, and the average particle size was 26.07 nm (Fig. 2A), which was in line with the particle size range specified by the MNEO. The particle size of MTEO was 375.54 nm, much larger than that of MNEO (Fig. 2B). The results show that the nanoemulsion had a good preparation effect, and the polymer dispersity index (PDI) was less than 0.1, which indicates that the particle size was very uniform and concentrated (Fig. 2C–D).

The light transmittance of MNEO treated at − 20 °C, 4 °C, 30 °C, 40 °C, and 50 °C was not significantly different from that of the control group (20 °C), indicating that different temperatures had little effect on the stability of MNEO (Figure S2A). The light transmittance at 1%, 2%, 3%, 4%, and 5% salt concentrations was not significantly different from that of the control group (Figure S2B). It is well known that salt will aggregate the particles in the nanoemulsion and have a negative impact^27^. MNEO was still stable at different salt concentrations, indicating that MNEO can resist available salt concentrations (Figure S2C). The nanoemulsion remained clear and transparent after centrifugation at different speeds for 10 min. The pH of MNEO was approximately 6.8, which was neutral. When the pH was adjusted between 5 and 9 by adding acid and alkali (Figure S2D), no stratification or precipitation was observed, which indicated stability. In the actual use of pesticides, as light is one of the most important factors affecting their activity, we measured light’s impact on MNEO and treated them at four different light intensities of 0%, 30%, 60%, and 90% for 48 h (Figure S2E–F). The results showed that after treatment, the appearance of MNEO was still clear and transparent. The antifungal effect had no significant change, indicating that the nanoemulsion had good light resistance.

The stability analysis mentioned above indicated that MNEO exhibited a smaller particle size than MTEO, meeting the necessary particle size specifications for a nanoemulsion. It has the advantages of high optical transparency, good physical properties, and prevention of gravity separation. No emulsification or phase separation was found in long-term storage, indicating that nanoemulsions were successfully prepared.

### Chemical component analysis of MEO, MTEO and MNEO

**Figure 3:**
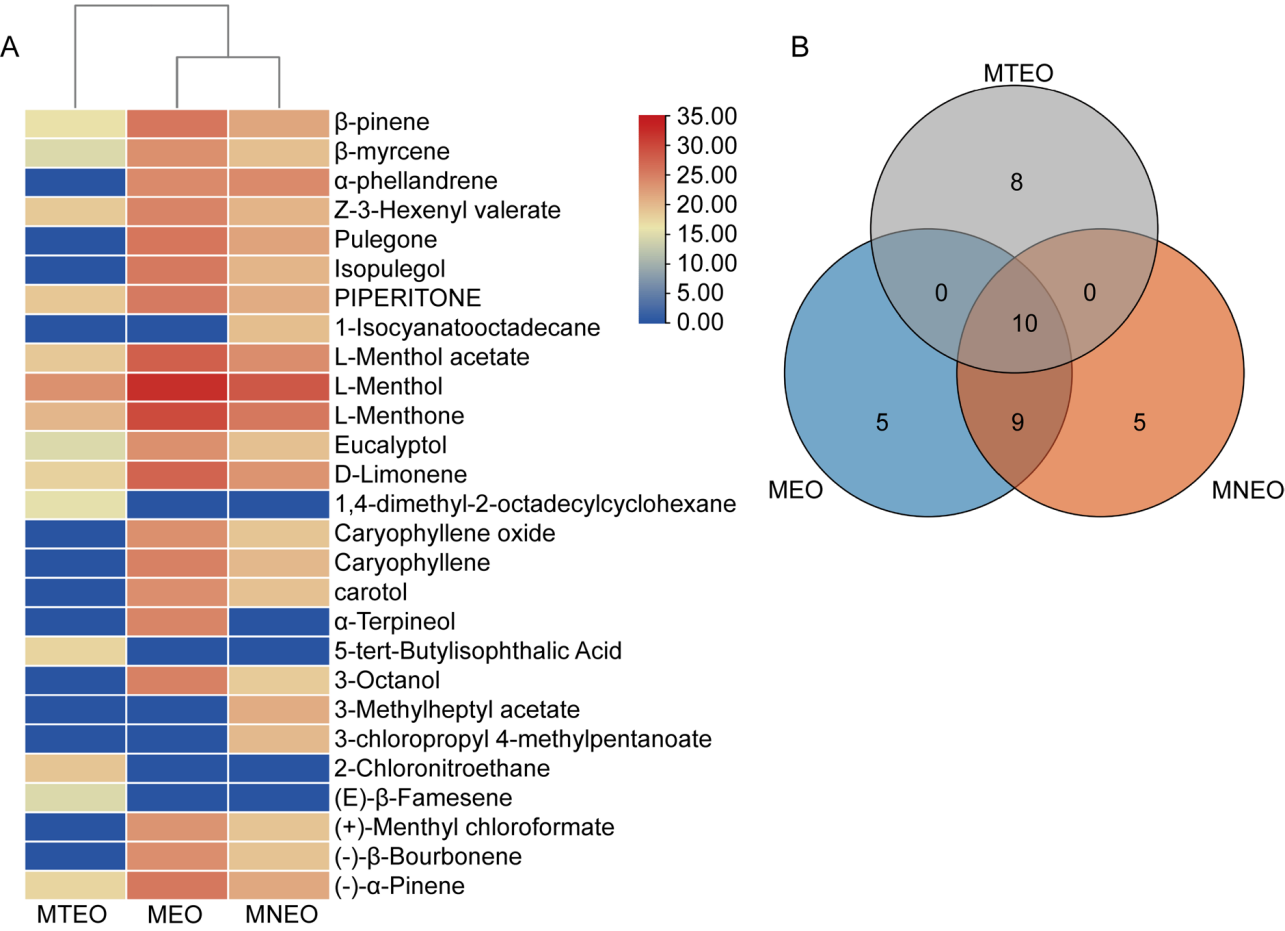
The chemical constituent analyses of MEO, MNEO, and MTEO were performed using GC‒MS. (**A**) Clustering heatmaps for each of the three groups using the base log scale of the GC‒MS peak area. (**B**) Venn diagram of the main chemical components in the three groups.

MEO has many beneficial biological functions, including antiviral, antioxidant, and antifungal effects^28^, and one of the most important parameters determining its antifungal activity is its chemical composition^17^. The chemical compositions of MEO, MTEO, and MNEO were analyzed by GC‒MS analysis. The total ion chromatograms were quantified using peak area normalization, and the relative percentage content of each chemical was calculated (Fig. 3). Twenty-four chemical components of MEO were identified, including l-Menthol (72.82%), l-Menthone (12.381%), l-Menthyl acetate (3.348%) and d-Limonene (2.386%). Our results were consistent with those of other studies where l-Menthol and l-Menthone were the most abundant components of MEO^29^.

Twenty-four chemical components were identified in MNEO, accounting for 98.29% of the emulsion content, with l-Menthol (77.852%), l-Menthone (10.429%), l-Menthyl acetate (2.848%) and d-Limonene (2.02%) being the main components. Compared with MEO, there were 19 common components with the same abundance and varieties, indicating that the nanoemulsion had little effect on the composition of MEO.

A total of 18 major compounds were identified in the MTEO, accounting for only 42.897% of the emulsion content, with most of the remainder being solvents. l-Menthol (32.294%) and l-Menthone (3.279%) accounted for the most significant proportion of the overall composition, with a greater chemical composition variation than MEO. Due to its poor solubility dispersion coefficient, traditional emulsions have resulted in only a tiny proportion of MEO being soluble in water.

### Inhibitory activity of MNEO and MTEO against *F. oxysporum*

**Figure 4:**
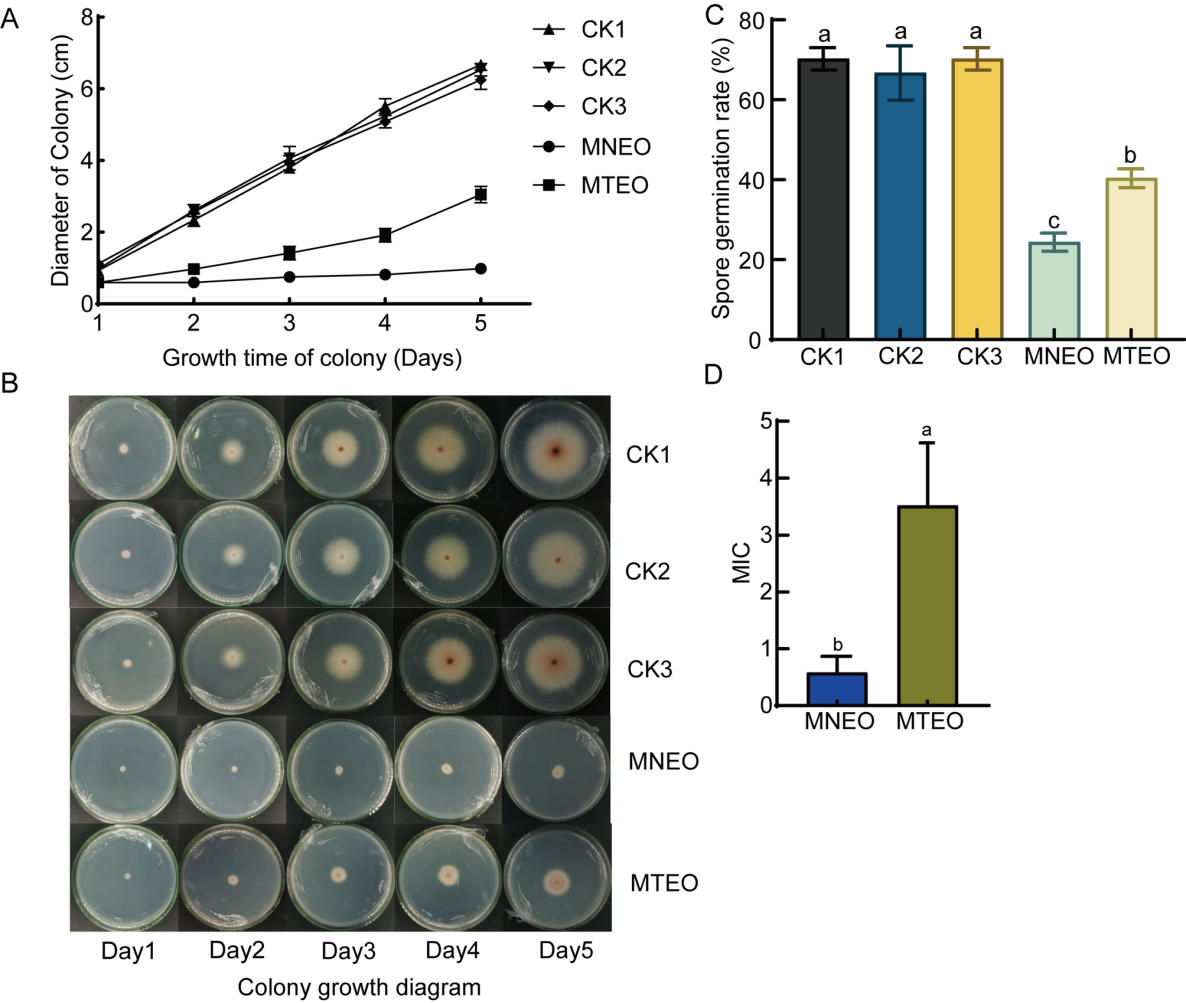
Determination of the antifungal activity of two emulsions against *F. oxysporum.* (**A**) Inhibitory effect of two emulsions on mycelial growth of *F. oxysporum*. (**B**) Colony growth of *F. oxysporum*. (**C**) Germination rate of spores. (**D**) The MIC of the two emulsions. CK1, An equal amount of sterile water was added to the medium; CK2, Solvents with nanoemulsions were added to the medium; CK3: Solvents of traditional emulsions were added to the medium, MNEO: *F. oxysporum* grown under MNEO treatment, MTEO: *F. oxysporum* grown under MTEO treatment. Data are presented as the means ± SDs of five biological replicates performed in triplicate.

The antifungal effect of the two emulsions on the growth of *F. oxysporum* was compared. At the same concentration (0.5 mg/mL), MNEO had the most significant effect on the growth of *F. oxysporum* colonies (Fig. 4A–B). The colony diameter did not change significantly within five days, while the colonies treated with MTEO were less affected and showed an increasing trend. The effects of the two solvents on the colony development of *F. oxysporum* were determined to test whether the selected solvents have toxic effects on colony growth. There was no significant difference in the growth of colonies with two solvent additions compared with the blank treatment group, indicating that the two solvents had no toxic effect (Fig. 4A).

The effects of the two emulsions on the spore germination rate of *F. oxysporum* were investigated at 0.5 mg/mL. After 15 h of culture, the germination rate of untreated spores was 64.78%, and the germination rate of MTEO-treated spores was 40.38%. At the same time, the spore germination rate after MNEO treatment was as low as 24.37% (Fig. 4C). The MICs of the two emulsions against *F. oxysporum* were 0.58 mg/mL and 3.51 mg/mL, respectively, as shown in Fig. 4D.

In conclusion, the antifungal activity of MNEO is greatly improved, which can more effectively inhibit the growth of *F. oxysporum* and improve its bioavailability.

### Effects of gene expression in *F. oxysporum* following treatment with MNEO and MTEO

**Figure 5:**
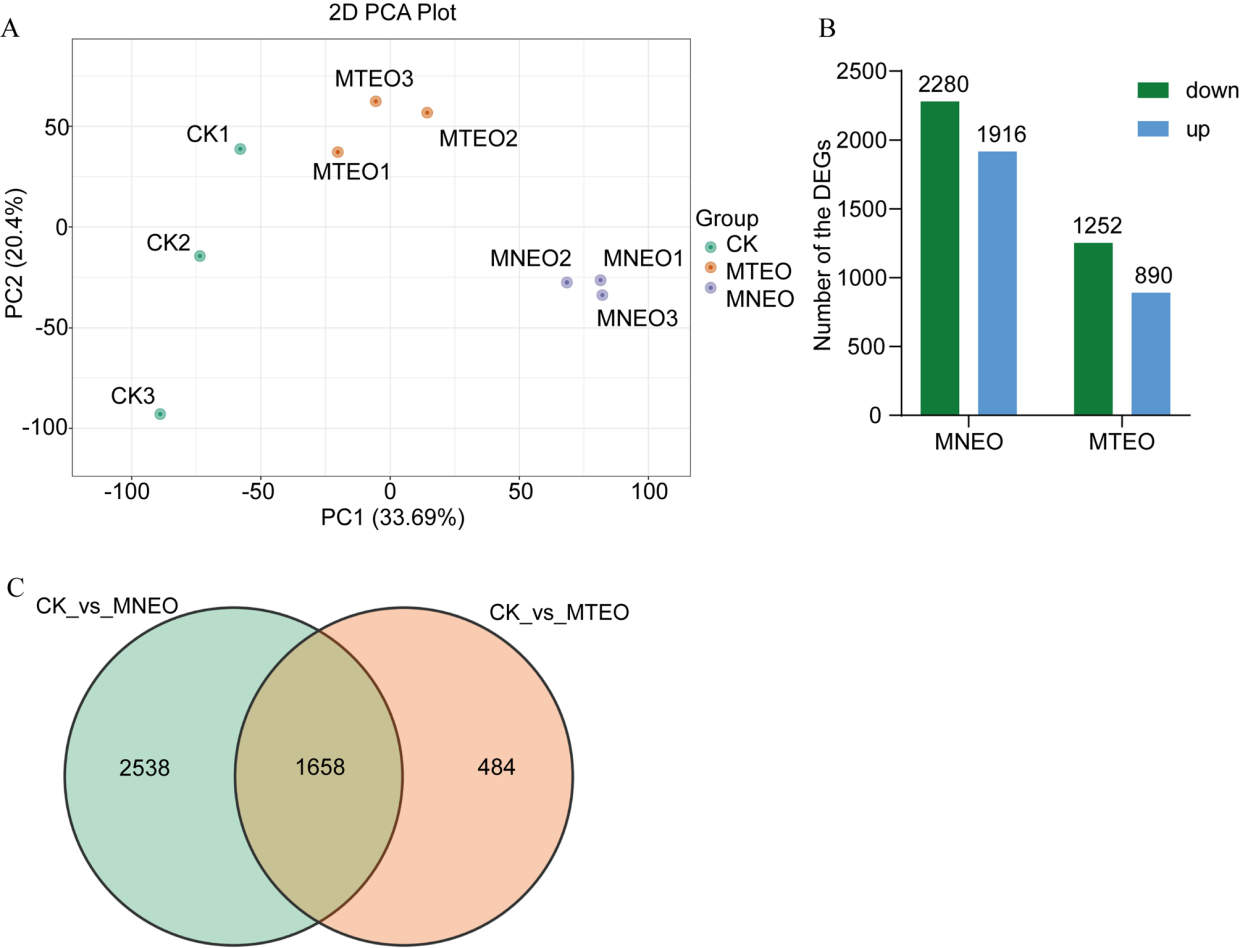
Transcriptomic analysis of *F. oxysporum* with MNEO and MTEO treatment. (**A**) Principal component analysis (PCA) of transcriptome data. (**B**) Upregulated and downregulated DEGs in the MNEO and MTEO treatment groups. (**C**) Venn diagram showing shared and unique DEGs of the MNEO and MTEO transcriptomes. Each treatment was the same as above.

Previous studies found that MNEO can inhibit the mycelial growth and spore germination of *F. oxysporum*. To study the mechanism of action of MNEO (0.5 mg/mL) on *F. oxysporum*, we evaluated the transcriptome changes of *F. oxysporum* after treatment with two formulations of MEO. PCA preliminarily verified the differences between CK, MNEO, and MTEO. The trend of separation and distinction between groups was evident, and the reproducibility of samples in the group was good (Fig. 5A).

Based on the differential expressed genes (DEGs) analysis of *F. oxysporum* RNA-seq between MNEO treatment and control, 4196 DEGs were identified, of which 1916 genes were upregulated and 2280 were downregulated. Compared to the control, 2142 DEGs were identified in the MTEO treatment group, of which 1250 genes were downregulated and 89 were upregulated (Fig. 5B). Under the same concentration treatment, MNEO treatment had a greater impact on the transcriptome of *F. oxysporum* (Fig. 5C). Then, KEGG enrichment analyse was performed on the DEGs to explore the antifungal mechanism of the two emulsions and further clarify the advantages of the MNEO treatment group at the molecular level.

### KEGG analysis between MNEO and MTEO treatment in transcriptomics

**Figure 6:**
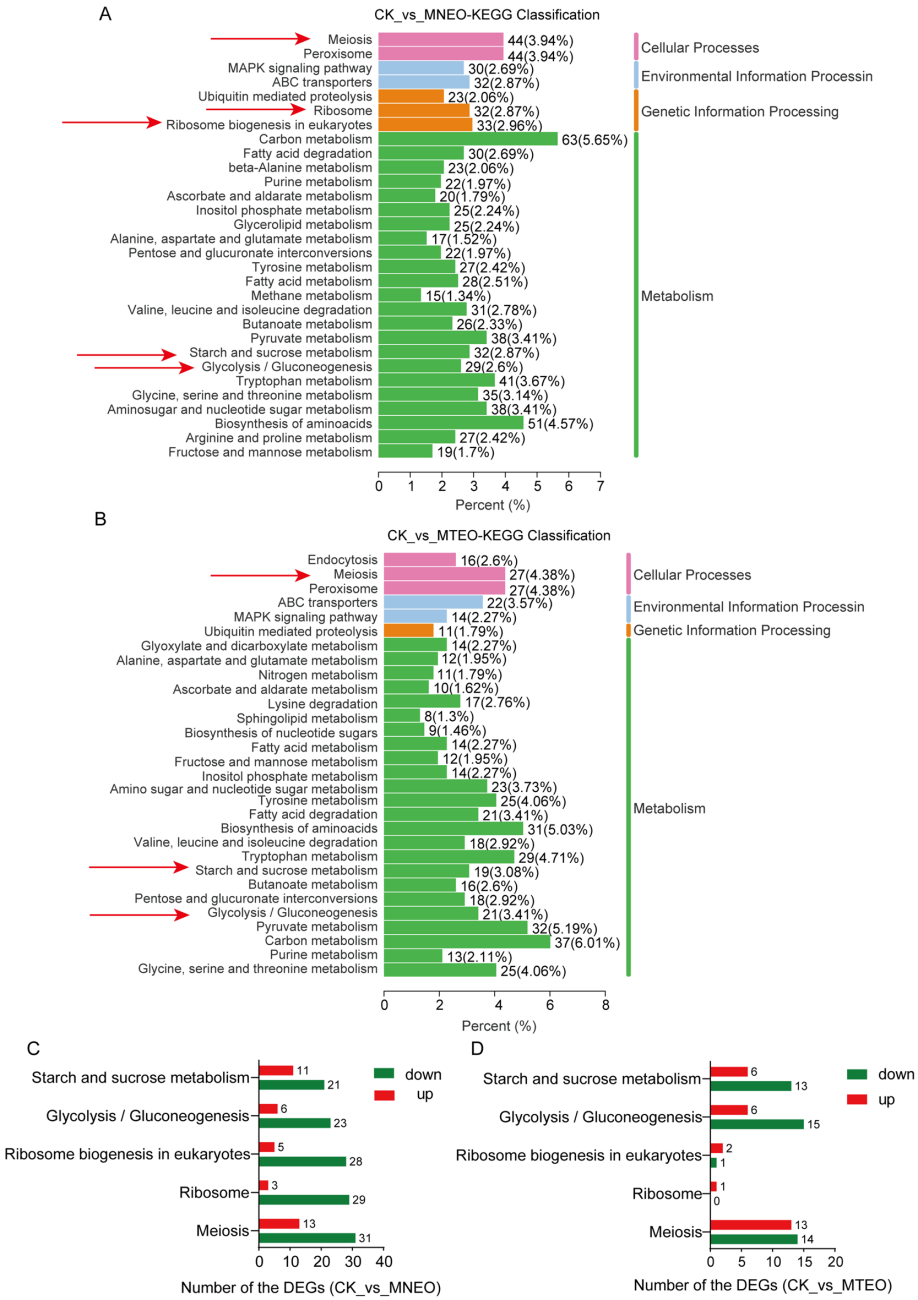
KEGG pathway enrichment analysis of DEGs in *F. oxysporum* exposed to MNEO (**A**) and MTEO (**B**). Upregulated and downregulated DEGs in 5 biological metabolic pathways shared by MNEO treatments (**C**) and MTEO (**D**).

KEGG pathway enrichment analysis showed that many pathways were significantly enriched in MNEO and MTEO compared to CK. In the early stage of the experiment, we found that the two emulsions could inhibit the growth and spore germination of *F. oxysporum*. Therefore, we further analyzed the biological pathways involved in these phenotypes. By analyzing the top 20 biological pathways, the meiosis, glycolysis/gluconeogenesis, and starch and sucrose metabolism pathways were directly related to fungal growth and development (Fig. 6A–B).

Most of the genes encoding major facilitator superfamily (MFS) transporter, speckled protein (SP) family, sugar: H^+^ symporter (HXT), and guanine nucleotide-binding protein G(i) subunit α (GNAI) in the meiosis pathway were significantly downregulated (Table S1). In addition, the gene difference was more significant in the MNEO treatment group; GNAI and GTPase Kras (KRAS) were significantly downregulated. Further analysis showed that the genes encoding cyclin-dependent kinase (CDC28), meiosis-specific transcription factor NDT80, and anaphase-promoting complex subunit 6 (APC6) were significantly downregulated after MNEO treatment in the middle and late stages of meiosis. In summary, this may be one of the reasons why MNEO treatment has a more substantial inhibitory effect on *F. oxysporum*.

Most DEGs were downregulated in the glycolysis/gluconeogenesis and starch and sucrose metabolism pathways (Fig. 6C–D). For example, genes encoding active enzymes such as fructose-bisphosphate aldolase, class II (ALDO), triosephosphate isomerase (TIM), phosphoglycerate kinase (PGK), and 2,3-bisphosphoglycerate-independent phosphoglycerate muta (gpmI) in the glycolysis/gluconeogenesis pathway are downregulated. In addition, MNEO inhibited the expression of more genes encoding aldehyde dehydrogenase NAD^+^ (ALDH). Additionally, MNEO inhibited the expression of genes encoding phosphoenolpyruvate carboxykinase (pckA) and glyceraldehyde 3-phosphate dehydrogenase (GAPDH), while there was no significant difference in the MTEO treatment group (Table S2). Similarly, the genes encoding 15 enzymes in starch and sucrose metabolism were significantly downregulated, seven unique to the MNEO treatment group (Table S1).

Finally, ribosome and ribosome biogenesis in eukaryotes are pathways for DEG enrichment only under MNEO stress. Most of the DEGs in the two metabolic pathways were downregulated (Fig. 6C–D), indicating that MNEO had a strong inhibitory effect on these pathways. In the ribosomal pathway, MNEO mainly inhibited the 29 genes encoding S26e, S16, L26e, and other ribosomal proteins (Fig. 6C, Table S2). A total of 28 genes encoding enzymes were downregulated in ribosome biogenesis in eukaryotes, such as GTPase1, RMP1, BMS1, POP4, and NOB1 (Fig. 6C, Table S2).

These results indicate that DEGs involved in the above pathways may be more closely related to the response of *F. oxysporum* to MNEO and MTEO stress. Furthermore, the stress effect of MNEO on *F. oxysporum* is more substantial, which confirms that MNEO has a more potent antifungal effect than MTEO.

### Metabolic changes in* F. oxysporum* under the MNEO and MTEO treatment

**Figure 7:**
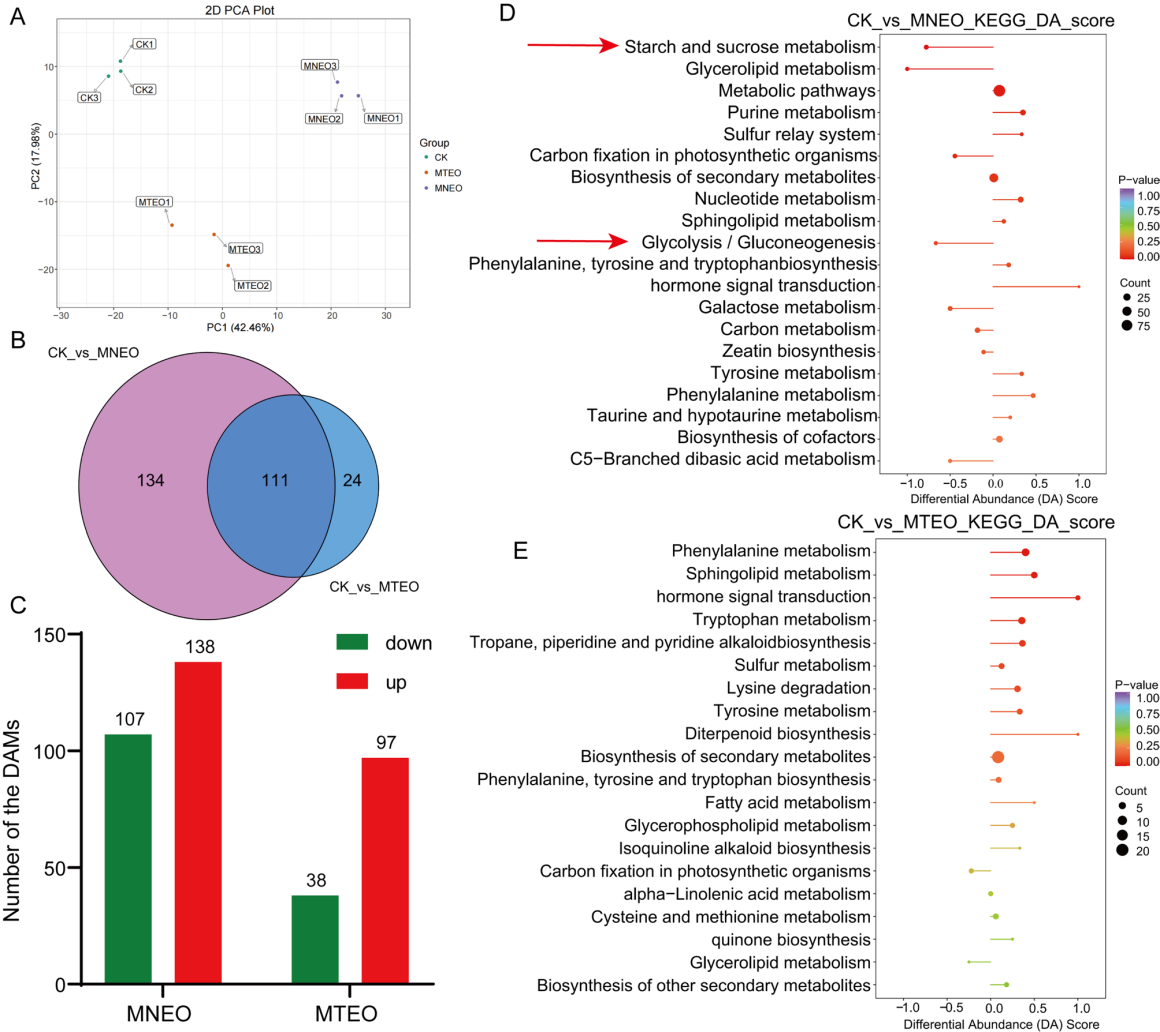
Extensive targeted metabolomic analysis of *F. oxysporum* following MNEO and MTEO treatment. (**A**) Metabolite PCA plots. (**B**) Wayne plots of DAMs between MNEO and MTEO treatment. (**C**) The number of upregulated and downregulated DAMs under MNEO and MTEO stress. Metabolomics reveals the enrichment pathway of MNEO (**D**) and MTEO (**E**) inhibition of *F. oxysporum* growth. A DA score > 0 indicates upregulation of all identified metabolites within the pathway, while a DA score < 0 indicates downregulation.

The metabolomics of the two treatment groups was analyzed to understand the antifungal mechanism of MNEO and MTEO on *F. oxysporum*. PCA results showed that PC1 explained 42.46% of the total variance. PC2 explained 17.98% of the total variance, with good separation between the treatment groups (Fig. 7A). There were 245 differentially accumulated metabolites (DAMs) in the MNEO treatment group and 135 DAMs in the MTEO treatment group. The intersection and specificity of metabolites in different subgroups were analyzed using Wayne diagrams. The number of DAMs shared in several differential subgroups was 111, and the number of differential metabolites specific to each differential subgroup was 134 and 24, respectively (Fig. 7B). The numbers of upregulated and downregulated metabolites in the MNEO and MTEO treatment groups were 138 and 107 and 97 and 38, respectively (Fig. 7C).

The DAMs were categorized, with the MNEO treatment consisting of amino acids and derivatives (18.37%), phenolic acid (16.73%), nucleotides and organic derivatives (12.24%), organic acids (11.84%), and other types of metabolites (Figure S2A). The MTEO treatment was primarily composed of phenolic acids (20.74%), amino acids and derivatives (17.04%), organic acids (11.85%), and other types of metabolites (Figure S2B).

KEGG enrichment analysis of these metabolites revealed that DAMs in the MNEO-treated group were in the starch and sucrose metabolism, glycolysis/gluconeogenesis, and purine metabolism pathways. Starch and sucrose metabolism and glycolysis/gluconeogenesis were also enriched in the transcriptome; most of these differential metabolites were downregulated (DA score < 0), and there were differences in 6 and 7 metabolites, respectively (Fig. 7D, Figure S2C–D). In the MTEO treatment group, DAMs were mainly enriched in phenylalanine metabolism, sphingolipid metabolism, tryptophan metabolism, and other pathways. Amino acid metabolism pathways accounted for a large proportion, and most DAMs were differentially upregulated (DA score > 0) (Fig. 7E). However, in the MTEO-treated group, no DAMs were identified in the starch and sucrose metabolic pathways, and only two DAMs, d-glycerate-3-phosphate (d-Glycerate-3P) and d-fructose-1,6-bisphosphate (d-Fructose-1,6P_2_) were identified in the glycolysis/gluconeogenesis pathway. These findings indicate that the impact of MTEO on the accumulation of metabolites in these two pathways was not statistically significant (Figure S2C).

In summary, we found more differential metabolites in the MNEO treatment group through metabolomics analysis, mainly manifested in the downregulation of significantly enriched pathways (top 20). Starch and sucrose metabolism and glycolysis/gluconeogenesis were also significantly enriched, and the accumulation of multiple metabolites decreased, but these two pathways were not enriched in the MTEO treatment group. The DAMs enrichment pathways in the MTEO treatment group were mainly upregulated and most related to fungal stress resistance. This also shows that MNEO is more destructive to *F. oxysporum.*

### Integrated transcriptomics and metabolomics analyses

Through transcriptome and metabolomics analysis, we found that MNEO treatment significantly suppressed glycolysis/gluconeogenesis and starch and sucrose metabolism. In the presence of MNEO, 7 DAMs (6 downregulated) and 32 DEGs (11 upregulated and 21 downregulated) were identified in the starch and sucrose metabolism of *F oxysporum,* while 6 DAMs (6 downregulated) and 29 DEGs (6 upregulated and 23 downregulated) were identified in the glycolysis/gluconeogenesis pathway. However, there was no significant enrichment of metabolites in these two pathways after MTEO treatment. Therefore, a combined analysis of DEGs and DAMs involved in glycolysis/gluconeogenesis and starch and sucrose metabolism was conducted for the MNEO treatment group.

**Figure 8:**
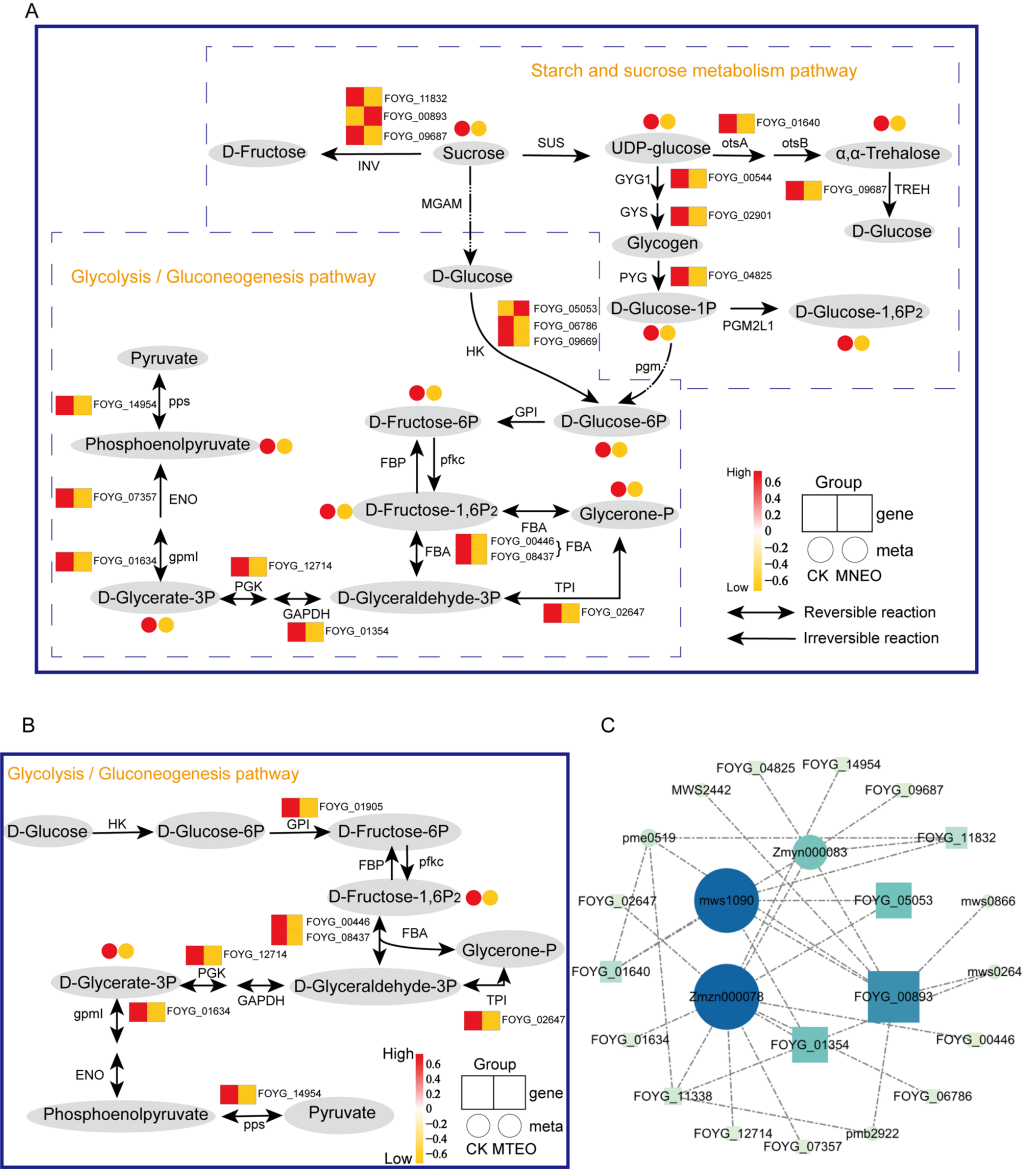
Combined transcriptomic and metabolomic analysis of glycolysis/gluconeogenesis and starch and sucrose metabolis (**A**) DEGs and DAMs involved in glycolysis/gluconeogenesis and starch and sucrose metabolism in the MNEO treatment group. (**B**) Combined transcriptomic and metabolomic analysis of glycolysis/gluconeogenesis in the MTEO treatment group. d-Glucose-1P: d-glucose-1-phosphate, d-Glucose-1,6P_2_: d-glucose-1,6-bisphosphate, d-Glucose-6P: d-glucose-6-phosphate, d-Fructose-6P: d-fructose-6-phosphate, d-Fructose-1,6P_2_: d-fructose-1,6-bisphosphate, Glycerone-P: glycerone phosphate, d-Glyceraldehyde-3P: d-glyceraldehyde-3-phosphate, d-Glycerate-3P: d-glycerate-3-phosphate, SUS: sucrose synthase, otsA: trehalose 6-phosphate synthase, TREH: α, α-trehalase, GYG1: glycogenin, GYS: glycogen synthase, PYG: glycogen phosphorylase, PGM2L1: glucose-1,6-bisphosphate synthase, pgm: phosphoglucomutase, GPI: glucose-6-phosphate isomerase, INV: beta-fructofuranosidase, HK: hexokinase, pfkc: ADP-dependent phosphofructokinase/glucokinase, FBA: fructose-bisphosphate aldolase, class II, TPI: triosephosphate isomerase, GAPDH: glyceraldehyde 3-phosphate dehydrogenase, PGK: phosphoglycerate kinase, gpmI: 2,3-bisphosphoglycerate-independent phosphoglycerate mutase, ENO: enolase, pps: pyruvate, water dikinase. After annotation, the DEGs and metabolites were presented as heatmaps at the corresponding locations, with yellow (low) and red (high) scales. (**C**) Coexpression network of DGEs and metabolites involved in glycolysis/gluconeogenesis and starch and sucrose metabolism. The higher the degree of connection, the deeper the color of the circle, and the more remarkable.

MNEO inhibits the metabolism of sucrose and UDP-glucose in *F. oxysporum*, affecting subsequent glycogen synthesis and metabolism and trehalose synthesis. UDP-glucose is synthesized through trehalose 6-phosphate phosphatase (otsA) and α, α-trehalose, in which the gene encoding otsA is downregulated (Fig. 8). During the synthesis and decomposition of glycogen, the accumulation of d-glucose-1-phosphate (d-Glucose-1P), d-glucose-1,6-bisphosphate (d-Glucose-1,6P_2_), and d-glucose-6-phosphate (d-Glucose-6P) decreased consistently with the coding of glycogenin (GYG1). The related glycogen phosphorylase (PYG) genes were consistent (Fig. 8A).

The accumulation of d-Glucose-1P, d-Fructose-1,6P_2_, and d-Glucose-6P was reduced in the glycolysis/gluconeogenesis pathway (Fig. 8A). d-glucose is catalyzed by hexokinase (HK), glucose-6-phosphate isomerase (GPI), and ADP-dependent phosphofructokinase/glucokinase (pfkc) to synthesize d-Fructose-1,6P_2_, which provides the material basis for the subsequent glycolysis process. As shown in the figure, the gene encoding HK was downregulated, consistent with the decrease in the content of the intermediate substances d-fructose-6-phosphate (d-Fructose-6P) and d-Fructose-1,6P_2_. Under the action of fructose-bisphosphate aldolase (FBA), triosephosphate isomerase (TPI), glyceraldehyde-3-phosphate dehydrogenase (GAPDH), and phosphoglycerate kinase (PGK), d-Fructose-1,6P_2_ is decomposed into d-Glycerate-3P. The genes encoding these enzymes were downregulated, and the metabolites glycerone phosphate (Glycerone-P) and d-Glycerate-3P were also downregulated. Finally, the upstream gene encoding 2,3-bisphosphoglycerate-independent phosphoglycerate mutase ((gpmI) and enolase (ENO) of phosphoenolpyruvate was downregulated, consistent with its content reduction. The key genes of glycolysis/gluconeogenesis and starch and sucrose metabolism were found to be inhibited to varying degrees in the MNEO and MTEO treatment groups. In contrast, the number of DEGs in the MNEO treatment group was more affected. Moreover, the differential metabolites of MTEO were only d-Glycerate-3P and d-Fructose-1,6P_2_ (Fig. 8B). This shows that the MNEO treatment group has a more substantial inhibitory effect on this pathway, thus having better antifungal activity.

A coexpression network showed that 5 DEGs highly contributed to starch and sucrose metabolism and glycolysis/gluconeogenesis, including *FOYG_00893*, *FOYG_01354*, *FOYG_05053*, *FOYG_11832*, and *FOYG_11338*, which might highly contribute to carbohydrate metabolism under MNEO stress. In addition, differential metabolites, including Glycerone-P (Zmzn000078), d-Glucose-1P (mws1090), and d-Glucose-1,6P_2_ (Zmyn000083), were also key nodes in these pathways and should be further studied (Fig. 8C).

### Effectiveness of MNEO against root rot of *P. notoginseng*

**Figure 9:**
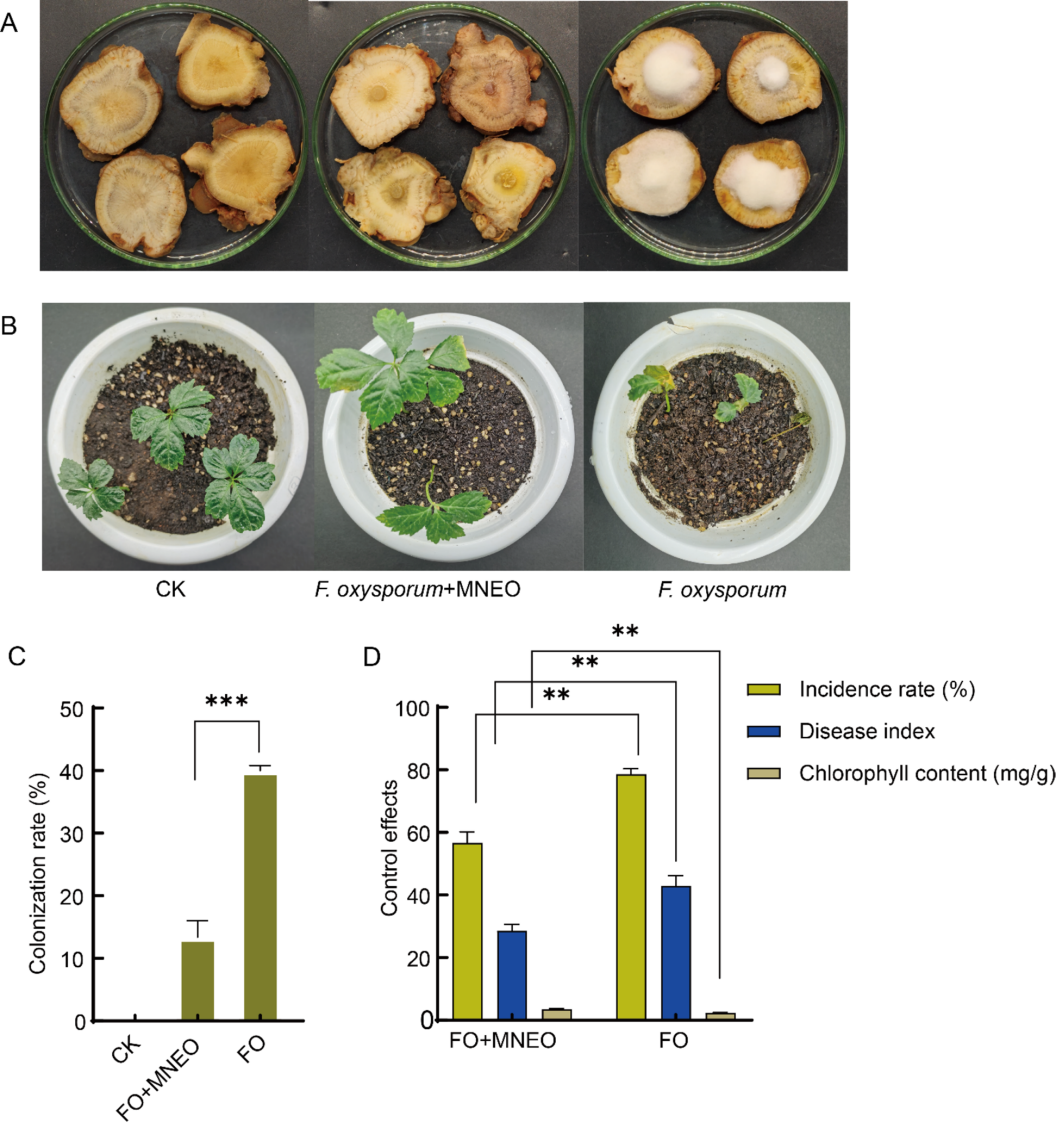
Effect of MNEO on controlling *F. oxysporum* infestation in *P. notoginseng* seedlings and isolated roots. (**A**, **C**) Effect of MNEO on the control of *P. notoginseng* isolated roots. (**B**, **D**) Effect of MNEO on the control of root rot of *P. notoginseng* seedlings caused by *F. oxysporum*. Data are presented as the means ± SDs of five biological replicates performed in triplicate. ***, *P* < 0.01. **, 0.01 < *P* < 0.05.

MNEO exhibited strong inhibition against *F. oxysporum* in the early stage of the experiment, and we assessed its impact on controlling root rot in *P. notoginseng* caused by *F. oxysporum*. The roots inoculated with *F. oxysporum* showed clear signs of decay, indicating its ability to survive in *P. notoginseng* roots and cause root rot (Fig. 9A). After infection with *F. oxysporum*, the incidence rate of *P. notoginseng* roots treated with MNEO was 13.41%, representing a significant decrease of 26.62% in incidence rate (Fig. 9C). The addition of the nanoemulsion significantly inhibited the growth of the fungal masses and reduced the infection rates and root rot occurrence.

The same results were found in the *P. notoginseng* seedlings (Fig. 9B), where seedlings infected with only the conidial solution of *F. oxysporum* had a high disease incidence of 78.63%. The disease index and chlorophyll content were 42.93 and 2.39 mg/g, respectively. After applying MNEO, the number of leaf discolorations was significantly reduced. The disease incidence was reduced by 21.91%. The disease index and chlorophyll content of *P. notoginseng* seedlings were 28.61 and 3.53 mg/g, respectively (Fig. 9D). The results of both experiments indicate that MNEO is effective in the suppression of *F. oxysporum* and the control of root rot of *P. notoginseng* and has the potential to be developed as a green pesticide.

### Verification of the transcriptome reliability using RT-qPCR

In order to verify the accuracy and reproducibility of the transcriptome analysis results, 9 genes related to sugar metabolism, ribosomes, and meiosis metabolic pathway were randomly selected and confirmed by RT-qPCR. For all 9 genes, RT-qPCR analysis revealed the same expression trends as the RNA-Seq data (Figure S2). It shows that the expression data obtained by RNA-Seq in this study is reliable.

## Discussion

The practical application of MEO as a fungal biocontrol agent for *P. notoginseng* root rot is limited by its poor water solubility, low bioavailability, and high volatility. Nanotechnology has been used to address these challenges by creating nanoemulsions to encapsulate and deliver EOs. Studies have shown that nanoemulsions can improve the bioavailability of plant EOs by helping them enter microbial cells and disrupting cell structure, thus enhancing their antifungal activity^30^. In this study, the prepared MNEO using the ultrasonic method has small homogeneous emulsion particle size (Fig. 2), good water solubility, and stability under different conditions (temperature, light, pH) without compromising its antifungal activity (Figure S2), consistent with previous reports^31^. In addition, compared to MTEO, the MNEO is water-miscible and exhibits a 14.41-fold reduction in particle size (Fig. 2), along with a 6.05-fold decrease in MIC and a 16.01% reduction in spore germination (Fig. 4). These results demonstrate that the nanoemulsion enhances the dispersion efficiency and stability of MEO, thereby improving its antifungal activity and bioavailability.

Plant EOs have complex chemical compositions influenced by a multitude of factors, including plant species, growth conditions, collection sites, and processing methods, which directly affect their antifungal activity^28^. Previous studies using GC–MS analysis identified l-Menthyl acetate and d-Limonene as the main components in MEO from *M. haplocalyx*^32^, consistent with our GC–MS results (Fig. 3). The relative proportion of these main components remains unchanged in MNEO after conversion into nanoemulsion, suggesting excellent inclusiveness that enhances the antifungal efficacy of MEO. l-menthol has been shown to inhibit *Candida albicans*, *Aspergillus niger,* and other fungi, while l-Menthol demonstrates strong inhibition against *Fusarium* and *Rhizobium*^33^. The enhanced inhibition effect on *F. oxysporum* observed upon conversion into nanoemulsion can be attributed to higher content of l-Menthol, l-Menthone, and l-Menthyl acetate in MNEO compared to MTEO, providing a theoretical basis for future development of antifungal products derived from MEO.

The biosynthesis and metabolism of glycogen are crucial for starch and sucrose metabolism, providing energy for vital life activities. GYG1 is essential for glycogen biosynthesis, while PYG regulates its decomposition by releasing d-Glucose-1P^34^. Inhibition of PYG reduces cell proliferation^35^. Our study found that both MNEO and MTEO suppress the gene expression of GYG1 and PYG, diminishing *F. oxysporum*’s energy source. Additionally, MNEO inhibits the gene expression of otsA, which synthesizes stress-protective trehalose using UDP-glucose^36^. Metabolomics analyses revealed that MNEO led to a significant decrease in the accumulation of seven metabolites associated with starch and sucrose metabolism in *F. oxysporum*, thereby disrupting energy metabolism processes and impeding the growth of the fungus (Figure S2). Notably, MNEO has stronger inhibitory effects on gene expression and metabolite accumulation than MTEO in this pathway (Fig. 6, Figure S2).

The accumulation of d-Glycerate-3P and d-Fructose-1,6P_2_ was mainly affected in glycolysis/gluconeogenesis by MNEO and MTEO treatment, leading to altered gene expression of FBA, TPI, PGK, and other essential enzymes for this pathway (Fig. 8). The decreased glycolytic synthesis of d-Glycerate-3P and reduced activity of the glycerolipid/free fatty acid (GL/FFA) cycle hinder cell growth by impeding lipid detoxification and critical metabolic intermediate production necessary for maintaining cellular homeostasis^37^. Lower levels of fungal spore formation and germination stages were observed at low concentrations of d-Fructose-1,6P_2_. The enzyme controlling the synthesis of d-Fructose-1,6P_2_, FBA, was identified as a potential inhibitory target for the natural fungicide iso butyryl benzene analog that inhibits *Magnaporthe grisea* growth^38,39^. Furthermore, exclusive MNEO treatment led to a significant downregulation of metabolites and genes such as d-Fructose-6P, phosphoenolpyruvate, *FOYG_01354,* and *FOYG_09920* (Figure S2 Table S1), indicating a more effective inhibition of energy metabolism in *F. oxysporum* by MNEO.

During meiosis, glucose is decomposed and converted through two main pathways, with their products utilized in each meiotic cycle. One pathway involves active transport through HXT into the membrane followed by GNAI-mediated decomposition to provide energy and material for subsequent meiotic processes^40^. The other pathway involves stimulation of cAMP synthesis through interaction with the G protein-coupled receptor (GPCR) Gpr1, GNAI, and glucose to generate substances and energy^41^. In this study, it was observed that both MNEO and MTEO inhibited the expression of genes encoding HXT and GNAI, impacting the activity of these enzymes, which may result in inadequate acquisition of actual energy, impeding normal meiosis and ultimately leading to slowed or halted fungal growth (Table S1). Furthermore, MNEO also significantly inhibits genes encoding key enzymes such as CDC28, NDT80, and APC6 (Table S1). These regulators play a role in meiosis, as well as in mitosis, DNA replication, and sexual development^42^.

Eukaryotic ribosome biosynthesis is fundamental to cellular life and is responsible for the regulation and coordination of many cellular processes, including cell growth and division^43,44^. Additionally, inhibiting eukaryotic ribosome biosynthesis can block mitochondrial respiration in *Beauveria bassiana*, disrupting energy metabolism and inhibiting strain growth to prevent fungal contamination^45^. In our study, following MNEO treatment, there was a significant inhibition of ribosome and ribosome biogenesis, leading to disruption of normal ribosomal function and impacting cell division and energy metabolism. Conversely, after MTEO treatment, genes associated with ribosome and ribosome biogenesis were not inhibited (Fig. 6, Table S2).

## Conclusion

**Figure 10:**
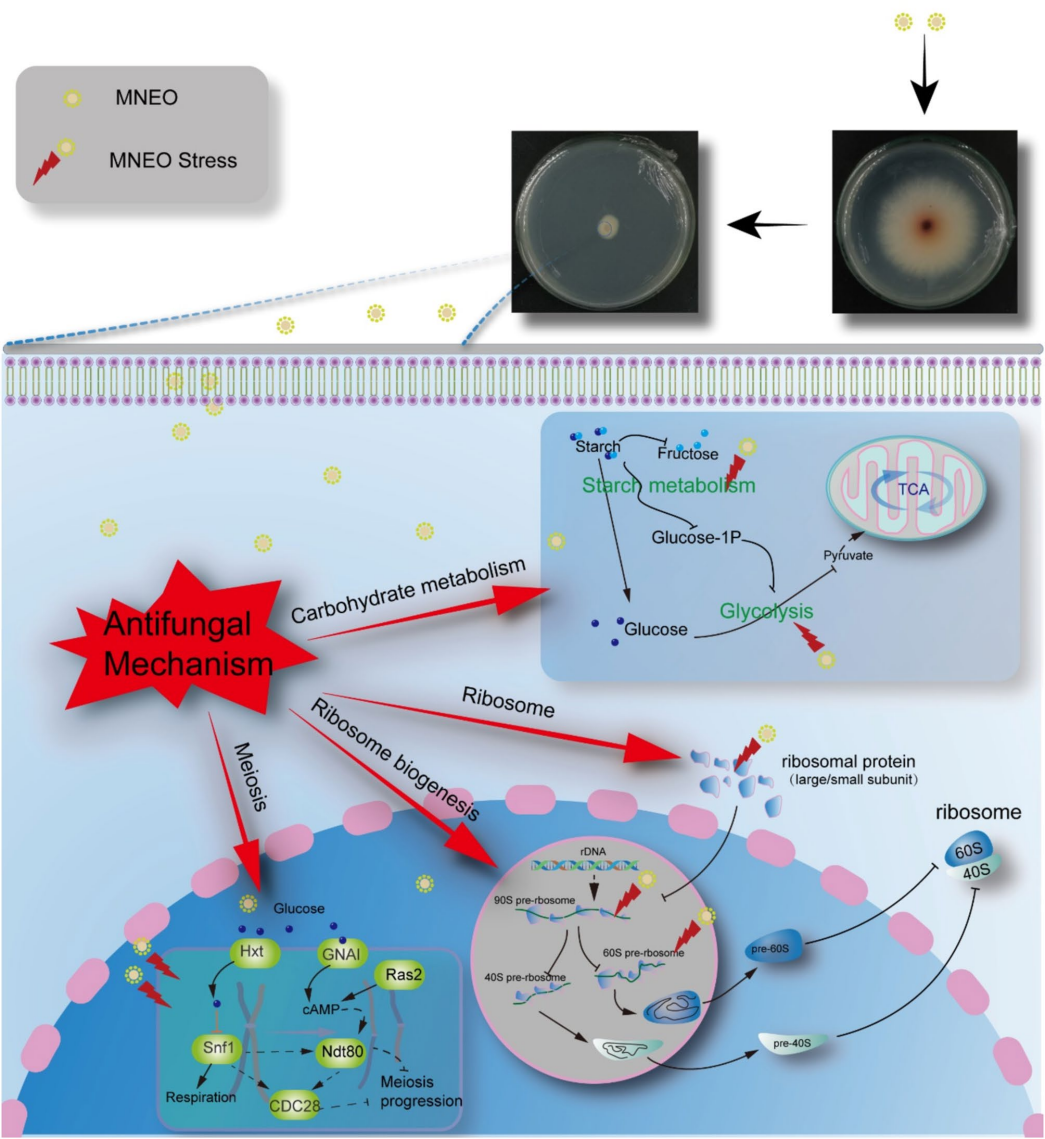
The mechanism model of the inhibitory effect of MNEO on *F. oxysporum* was analyzed from the perspective of multiomics. MNEO Stress indicates that the metabolic pathways or biological processes of *F. oxysporum* are inhibited after MNEO treatment.

In this study, an MEO nanoemulsion was successfully prepared using the ultrasonic method, effectively preserving key components of MEO such as l-Menthol, l-Menthone, and l-Menthyl acetate, thus demonstrating significant efficacy in inhibiting the growth of *F. oxysporum* in vitro. Transcriptome and metabolome analyses revealed that MNEO inhibition of *F. oxysporum* primarily impacts the expression and accumulation of genes and metabolites in three major categories: energy metabolism (starch and sucrose metabolism, glycolysis/gluconeogenesis), ribosomal function (ribosome, ribosome biogenesis), and cell reproduction (meiosis), including Glycerone-P, trehalose, UDP-glucose, and otsA, among others. Additionally, MNEO modulates gene expression related to meiosis, ribosome function, and biogenesis in eukaryotes to further inhibit the growth of *F. oxysporum* (Fig. 10). In practical applications, MNEO has shown promising outcomes. This study offers a theoretical framework for the further advancement and application of MEO in the development of eco-friendly pesticides and mitigation of root rot in medicinal plants.

## Materials and methods

### Fungus and plant material

The *P. notoginseng* root rot pathogen *F. oxysporum* was provided by the Wu Kai research group at Yunnan Normal University (GenBank: OQ080022.1). The strains were cultured on potato dextrose agar (PDA) medium and placed in a microbial incubator at 28 °C.

The seeds of *P. notoginseng* were purchased from Yunnan Wenshan Jinyuan Agricultural Development Co., Ltd. They were sown in a matrix mixed with nutrient soil: clay = 4:1 and cultured at a relative humidity of 65% ± 5% photoperiod for 14 h/d and a temperature of 20 °C until seed germination. MEO was purchased from Anhui Huatian Perfume Co., Ltd. Experimental research and field studies on the plants (either cultivated or wild), including the collection of the plant material are in compliance with relevant institutional, national, and international guidelines and legislation.

### Preparation of emulsion

#### Establishment of a nanoemulsion system for MEO

Surfactant (Tween 80) and cosurfactant (absolute ethanol) were accurately weighed in a beaker at a particular mass ratio (Km = 1, 2, 3, and 4). The ultrasonic treatment was carried out in an ice bath for 20 min with an operating power of 300 W. Then, select the best Km ratio. The total mass of the prepared mixed surfactant and MEO phase was 5 g, and the mass ratios were 9:1, 8:2, 6:4, 5:5, 4:6, 3:7, 2:8, and 1:9. Then, the mixture was allowed to stand at room temperature for 15 min, and distilled water was added dropwise with a 5 mL pipette, with stirring while dropping, and repeated until the solution changed from clear to turbid and back to clear. The mass fraction of the components at the transition point was calculated. The ternary phase diagram of the surfactant blend, MEO, and distilled water was drawn. The area was calculated to determine the optimal Km of the EO nanoemulsion system.

#### Preparation of MNEO

The surfactant (Tween 80) and the cosurfactant (anhydrous ethanol) were accurately weighed at the optimal mass ratio (Km = 3) determined in Sect. "Establishment of a nanoemulsion system for MEO" (Figure S1), and thoroughly mixed to obtain surfactant I. The surfactant I was then placed in a 50 mL beaker with the MEO at a ratio of 8:2, stirred magnetically for 10 min, and treated by ultrasound in an ice bath at 300 W for 20 min. Subsequently, deionised water was added in a ratio of 5:4 (oil phase: water phase), and the oil and water phases were stirred magnetically for 10 min. The resulting mixture was then sonicated in an ice bath at 300 W for 30 min (Fig. 1A).

#### Preparation of MTEO

Referring to the literature with slight modifications^46^, MEO was dissolved in surfactant II (aqueous solution of 2% DMSO and 0.1% Tween 80) according to the desired concentration, ultrasonicated in an ice bath for 20 min and set aside at 4 °C (Fig. 1B).

### Investigation of the appearance and stability of MNEO

#### Morphological observations and particle size analysis of the two emulsions

The two emulsions were diluted ten times with deionized water. For particle morphology, samples were observed under a transmission electron microscope (JEM-1011, JEOL, Co. Ltd., Tokyo, Japan). A laser particle size meter (90Plus PALS) was used to measure the particle size of the emulsions*.*

#### Centrifugation test

MNEO samples were prepared and centrifuged at 2000 r/min, 5000 r/min, and 8000 r/min for 30 min to observe if they were stratified, using distilled water as a blank control. The absorbance at 550 nm was measured before and after centrifugation.

$${\text{T}} = {1}0^{{ - {\text{A}}}}$$

T: Transmittance; A: Absorbance.

#### Salt stability

Five milliliters of MNEO were placed in 5 clean test tubes. Sodium chloride was added to them to make the mass fractions of sodium chloride 1%, 2%, 3%, 4%, and 5%, respectively, and the test tubes were shaken to dissolve. The changes in the nanoemulsion were observed. And use the same formula as above to calculate the transmittance.

#### Acid–base stability

Ten milliliters of MNEO were placed in a small beaker, and then 0.1 M hydrochloric acid or sodium hydroxide solution was gradually added dropwise to the cup. The pH was adjusted to acidic or alkaline, and the system’s pH change was measured using a pH meter. The difference in the nanoemulsion solution was observed, and use the same formula as above to calculate the transmittance.

#### The effect of temperature on the stability of MNEO

The prepared MNEO was heated at − 20 °C, 4 °C, 20 °C, 30 °C, 40 °C and 50 °C for 30 min to observe whether the nanoemulsions were delaminating; if not, the absorbance of the nanoemulsions before and after heating was measured, and use the same formula as above to calculate the transmittance.

#### Effect of light on antifungal activity of MNEO

The prepared MNEO was placed in a light incubator; the light intensity was set to 0%, 30%, 60%, or 90%; and the constant temperature was 25 °C for 48 h. After treatment, the antifungal effect was tested according to the method detailed in Section “Fungal growth curves”.

### Gas chromatography-mass spectrometry (GC‒MS) analysis

The prepared MTEO and MNEO were analyzed by GC‒MS using Agilent Technologies 7890B-5977B gas chromatography. The compounds’ retention times and mass spectra were compared with NIST 17.L database, and the final chemical constituents were determined with relevant literature^47^.

### Comparison of antifungal activity between MNEO and MTEO

#### Fungal growth curves

The MNEO, MTEO, sterile water, nanoemulsion solvent, and traditional emulsion solvent (the final emulsion concentration was 0.5 mg/mL) were added to 20 mL of PDA medium for five replicates. The PDA medium was inoculated with a 5 mm block of *F. oxysporum* and incubated at 28℃. The colony diameter was measured at 24 h intervals until the seventh day, and the growth curve of the two emulsions against *F. oxysporum* was obtained^48^.

#### Fungal spore germination rate

MNEO and MTEO were added to a sterile EP tube containing PDA liquid medium and mixed until the emulsion concentration was 0.5 mg/mL. Finally, an appropriate spore suspension was added to a final concentration of 1 × 10^6^ spores/mL, and the control group was added to sterile water. Each treatment was repeated three times^49^. The total number of spores and the number of germinated spores were counted by a blood cell counting plate under a light microscope.

$${\text{G }} = \, \left( {{\text{X}}_{{1}} /{\text{X}}_{0} } \right) \, \times { 1}00\%$$

G: spore germination rate; X_1_: number of spores that have germinated; X_0_: total number of spores.

#### Determination of MIC

The MIC value test was carried out with 96-well plates according to the reference with slight modification^50^. Fresh colonies were washed with 1/3 PDA liquid medium, and spore suspensions were obtained by filtration. The concentration was adjusted to 2 × 10^5^ spores/mL. The initial concentration of MNEO was 4 mg/mL, and MTEO was 25 mg/mL. Two emulsions are diluted into eight concentration gradients by double dilution. The 96-well plates were incubated at 28 °C for 20 h. A microplate reader measured the absorbance of each well at a wavelength of 595 nm, and the MIC value of the emulsion was calculated.

### Transcriptome and metabolomics analysis

#### Sample preparation

Emulsion at the concentration of 0.5 mg/mL was prepared for 30 mL, and then 1 g of *F. oxysporum* mycelium was added and treated at 28 °C for 24 h. The mycelium was harvested through filtration, rapidly frozen with liquid nitrogen, and stored at − 80 °C for subsequent use.

#### RNA extraction and RNA-Seq

Total RNA was extracted from the samples using the RNAprep pure plant kit (DP441, Tiangen, China). Illumina RNA-Seq was performed by Meisoft Biotechnology Co., Ltd. (Wuhan, China). RNA quality was measured by a nanophotometer spectrophotometer (IMPLEN, CA, USA), Qubit 2.0 fluorometer (Life Technologies, CA, USA) and Agilent Bioanalyzer 2100 system (Agilent Technologies, CA, USA). Poly (A) mRNA was enriched with oligomer (dT) magnetic beads and randomly fragmented mRNA. The first strand of cDNA was synthesized by the M-MuLV reverse transcriptase system. Then, the RNA strand was degraded with RNase H, and cDNA was synthesized with DNA polymerase. These double-stranded cDNAs were connected to the sequencing adapter. The cDNA (~ 200 bp) was screened with AMPure XP beads. After amplification and purification, the cDNA library was obtained and sequenced using the Illumina Novseq 6000 system^51^.

#### Sequence data processing

The original sequence of the original image data was converted by base recognition technology. To obtain high-quality data, the sequence joints were cut, and fastp was used to remove low-quality reads containing ≥ 5 uncertain bases or more than 50% Qphred ≤ 20 bases. The GC content of the clean read was calculated. Fast QC also generated Q20 and Q30 values to evaluate the essential quality. Then, clean reads were mapped to the *F. oxysporum* reference genome using HISAT2 with default parameters. Gene expression levels were determined using the RPKM (per million reads) method.

#### Extraction of metabolites

Biological samples were freeze-dried using a vacuum freeze dryer (Scientz-100F). The freeze-dried samples were ground with a mixer mill (MM 400, Retsch) and zirconia beads at 30 Hz for 90 s. Lyophilized powder (100 mg) was dissolved in 1.2 mL of 70% methanol solution, rotated for 30 s every 30 min six times, and placed in a refrigerator at 4 °C overnight. After centrifugation at 12,000 rpm for 10 min, the extract was subjected to UPLC‒MS/MS analysis (SCAA-104, 0.22 μm pore size; an NPEL, Shanghai, China, http://www.anpel.com.cn/).

#### Qualitative and quantitative analysis of metabolites

Metabolite data were log2 transformed for statistical analysis to improve normality and normalization. Hierarchical clustering analysis (HCA), principal component analysis (PCA), and orthogonal partial least squares discriminant analysis (OPLS-DA) were performed on the metabolites of 30 samples to study the specific accumulation of metabolites. The p-value and fold change values were set to 0.05 and 2.0, respectively. The Venn diagram illustrates the number of bidirectional metabolites. The Kyoto Encyclopedia of Genes and Genomes (KEGG) database was used, with a *p*-value < 0.01, and the standard database of *F. oxysporum* (String database species ID:5507) was referenced. All data were plotted using GraphPad Prism 99v6.01 (GraphPad Software, La Jolla, CA, USA)^51^.

### In vivo testing of the control effect

The roots of *P. notoginseng* with uniform size were selected and cut into approximately 2 cm thick pieces after cleaning. After disinfection with alcohol and sodium hypochlorite, the pieces were placed on a plate covered with water agar. The 5 mm diameter of the fungal blocks was positioned at the center of the *P. notoginseng* block and served as a positive control, while 5 μL of MNEO at a concentration of 0.5 mg/mL was added to the fungal block as a treatment. Following a seven-day incubation period, the growth of fungal blocks on *P.notoginseng* was quantified, and the incidence rate was calculated.

Uniformly grown annual *P. notoginseng* seedlings (10 cm high) were selected, and 10 mL of *F. oxysporum* suspension at a concentration of 1 × 10^6^ spores/mL was injected into the soil with a syringe around the base of the plant stems. Forty plants were planted in each treatment. MNEO at a 0.5 mg/mL concentration was prepared, and 10 mL was poured into the base of each plant stem every seven days, while 10 mL of sterile water was poured into the blank control. *P. notoginseng* was incubated in a 12 h light/12 h dark incubation chamber at 24 °C to observe disease development. The incidence and disease index of *P notoginseng* were recorded on the 30th day after the initial application of MNEO, and the chlorophyll content was determined using an established method^52,53^.

$${\text{Disease incidence }}\left( {{\text{DI}}} \right) \, = {\text{ number of diseased plants}}/{\text{total number of plants}} \times {1}00\%^{{{53}}} .$$

Disease severity was graded as follows.

- Grade 0: plants are healthy.
- Grade 1: spots on the leaves of the plants.
- Grade 2: wilting of the plant.
- Grade 3: plants are dead.
  $${\text{Disease index }}\left( {{\text{Di}}} \right) \, = {1}00 \times \sum \left( {{\text{Dn}} \times {\text{Dg}}} \right)/\left( {{\text{Tn}} \times {\text{Mg}}} \right)$$
  where Dn indicates the number of plants with the same disease level, Dg is the corresponding disease level, Tn is the total number of plants, and Mg is the highest disease level.

### RT‒qPCR analysis

The samples were taken from mycelia that were treated with different EOs (CK, MNEO, MTEO) for 24 h and used for RNA extraction. Each sample was reverse-transcribed into first strand cDNA using a cDNA reverse transcription kit (TaKaRa PrimeScript RT Master Mix RR036B). The primer list is shown in the supplementary table (Table S3). RT‒qPCR was performed using a ChamQ SYBR qPCR Master Mix (Vazyme) and a Bio-Rad CFX Manager 3.1 thermal cycle system. Relative transcript levels were calculated using the 2^−ΔΔCq^ method, using a housekeeping gene, QTUB, as a reference. Three biological and technical replications were performed.

### Statistical analysis

All experiments in this study were repeated three times, and the data shown are the mean ± SD (standard deviation). The results were subjected to one-way ANOVA (Pvalue of ≤ 0.05), LSD (least-significant difference), and Tukey’s multiple range test to determine significant differences in mean comparisons. These data were analyzed using the SPSS program version 19.0. The results were finally plotted using the Origin program version 2019b, Adobe Illustrator 2022, and GraphPad Prism 9.

### Supplementary Information

Supplementary Information.

## Data Availability

The Transcriptome sequencing data of this study were submitted and deposited in the NCBI GenBank database under BioProject No. PRJNA1076078. The datasets generated during and/or analysed during the current study are available from the corresponding author on reasonable request.
