# Supplementary material for: Study on the inhibitory activity and mechanism of Mentha haplocalyx essential oil nanoemulsion against Fusarium oxysporum growth
Source: Sci Rep. 2024 Jul 11;14:16064. doi: 10.1038/s41598-024-67054-1 (PMC11239933; doi:10.1038/s41598-024-67054-1)
Supplement: Supplementary file 1 — Supplementary Information. [file 41598_2024_67054_MOESM1_ESM.doc]

Study on the inhibitory activity and mechanism of *Mentha haplocalyx* essential oil nanoemulsion against *Fusarium oxysporum* growth

*Hongxin Liao, Jinrui Wen, Hongyan Nie, Cuiqiong Ling, Liyan Zhang, Furong Xu, Xian Dong^*^*

School of Chinese Materia Medica, Yunnan University of Chinese Medicine, Kunming 650000, China,

* Corresponding author: [dongxian_1655129@163.com](mailto:dongxian_1655129@163.com)

**Abstract:** *Mentha haplocalyx* essential oil (MEO) has demonstrated inhibitory effects on *Fusarium oxysporum*. Despite its environmentally friendly properties as a natural product, the limited water solubility of MEO restricts its practical application in the field. The use of nanoemulsion can improve bioavailability and provide an eco-friendly approach to prevent and control *Panax notoginseng* root rot. In this study, Tween 80 and anhydrous ethanol (at a mass ratio of 3) were selected as carriers, and the ultrasonic method was utilized to produce a nanoemulsion of MEO (MNEO) with an average particle size of 26.07 nm. Compared to MTEO (MEO dissolved in an aqueous solution of 2% DMSO and 0.1% Tween 80), MNEO exhibited superior inhibition against *F. oxysporum* in terms of spore germination and hyphal growth. Transcriptomics and metabolomics results revealed that after MNEO treatment, the expression levels of certain genes related to glycolysis/gluconeogenesis, starch and sucrose metabolism were significantly suppressed along with the accumulation of metabolites, leading to energy metabolism disorder and growth stagnation in *F. oxysporum*. In contrast, the inhibitory effect from MTEO treatment was less pronounced. Furthermore, MNEO also demonstrated inhibition on meiosis, ribosome function, and ribosome biogenesis in *F. oxysporum* growth process. These findings suggest that MNEO possesses enhanced stability and antifungal activity, which effectively hinders *F. oxysporum* through inducing energy metabolism disorder, meiotic stagnation, as well as ribosome dysfunction, thus indicating its potential for development as a green pesticide for prevention and control *P. notoginseng* root rot caused by *F.oxyosporum*.

**Keywords:** nanometer emulsion, ultrasonic method, antifungal mechanism, metabolism pathways, prevention of root rot.


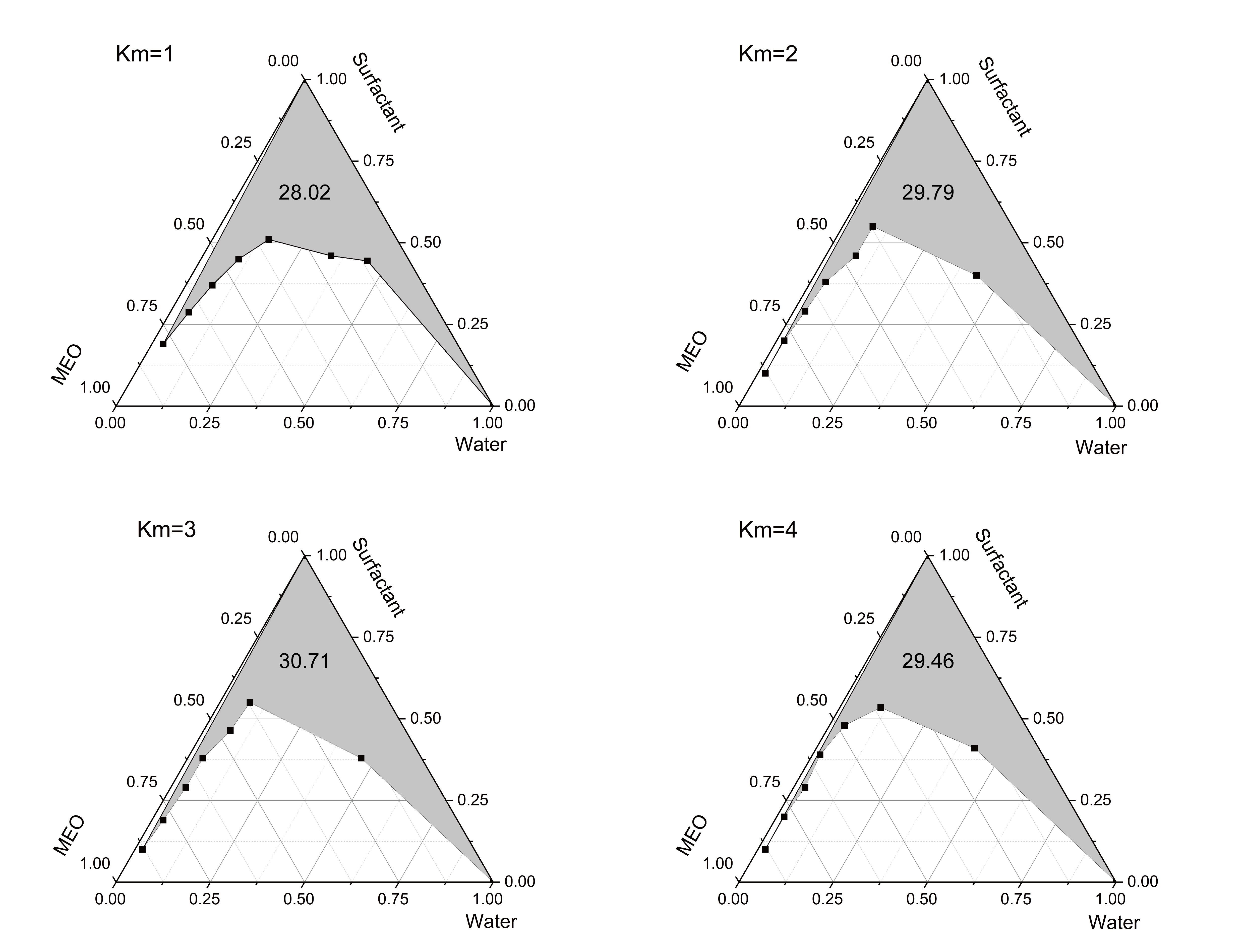


**Figure S1** The effect of mixing Tween 80 with anhydrous ethanol according to different Km values on the ternary phase diagram of the nanoemulsion. The area of the shaded part screened the best Km.


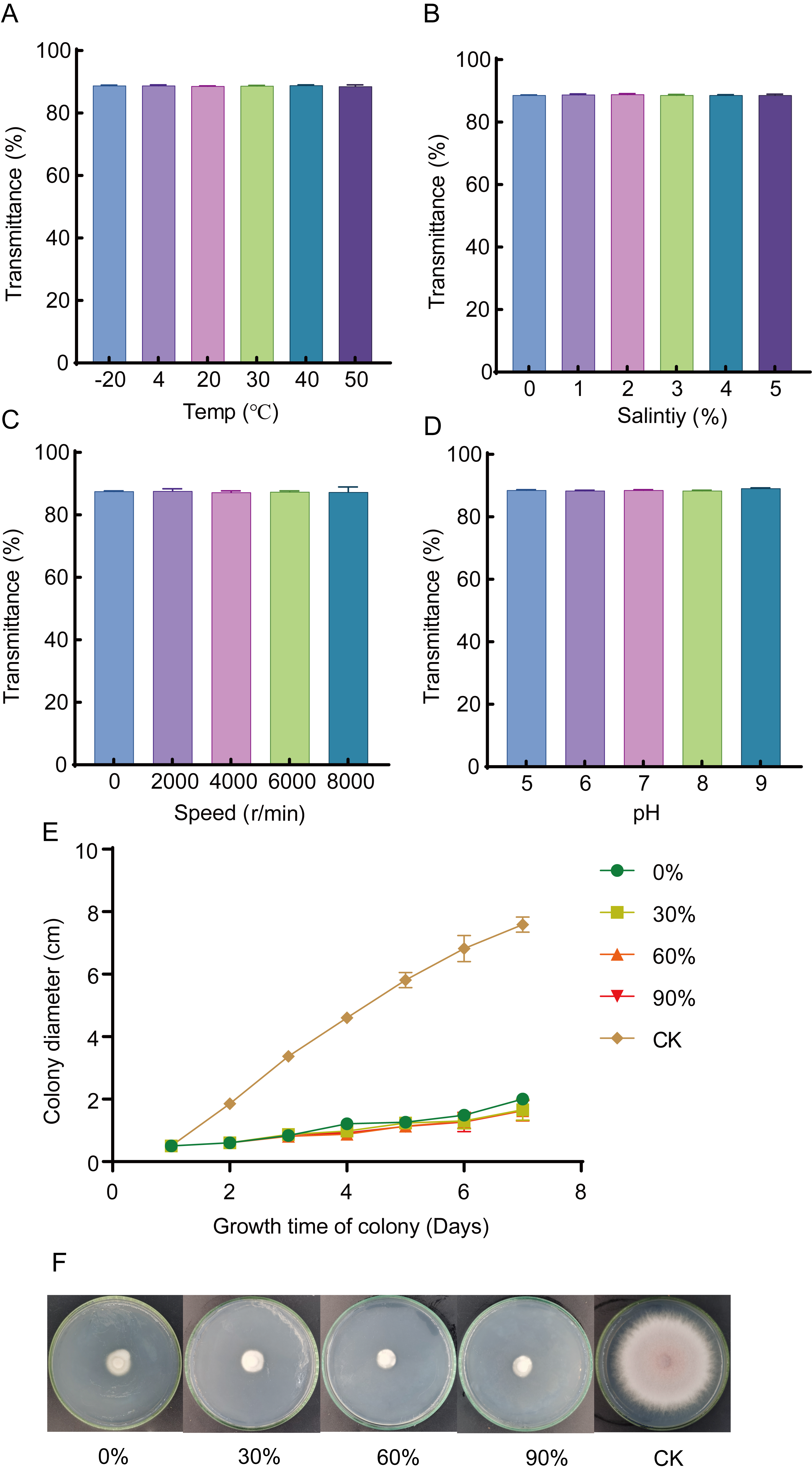


**Figure S2** Nanoemulsion stability testing. (A) Transmittance at different temperatures for 30 min. (B) The effect of varying salinity on the nanoemulsion. (C) Changes in the light transmittance of the nanoemulsion after centrifugation with different centrifugal forces for 30 min. (D) Changes in the light transmittance of the nanoemulsion after adjusting to different pH values with 0.2 M HCl and 0.2 M NaOH. (E) Effect of different light intensities on the stability and antifungal activity of MNEO after 48 h of treatment. (F) Pictures of colony growth on the seventh day, with 0%, 30%, 60%, and 90% representing different light intensities. CK was not treated with MNEO. Data are presented as the means ± SDs of three biological replicates performed in triplicate.


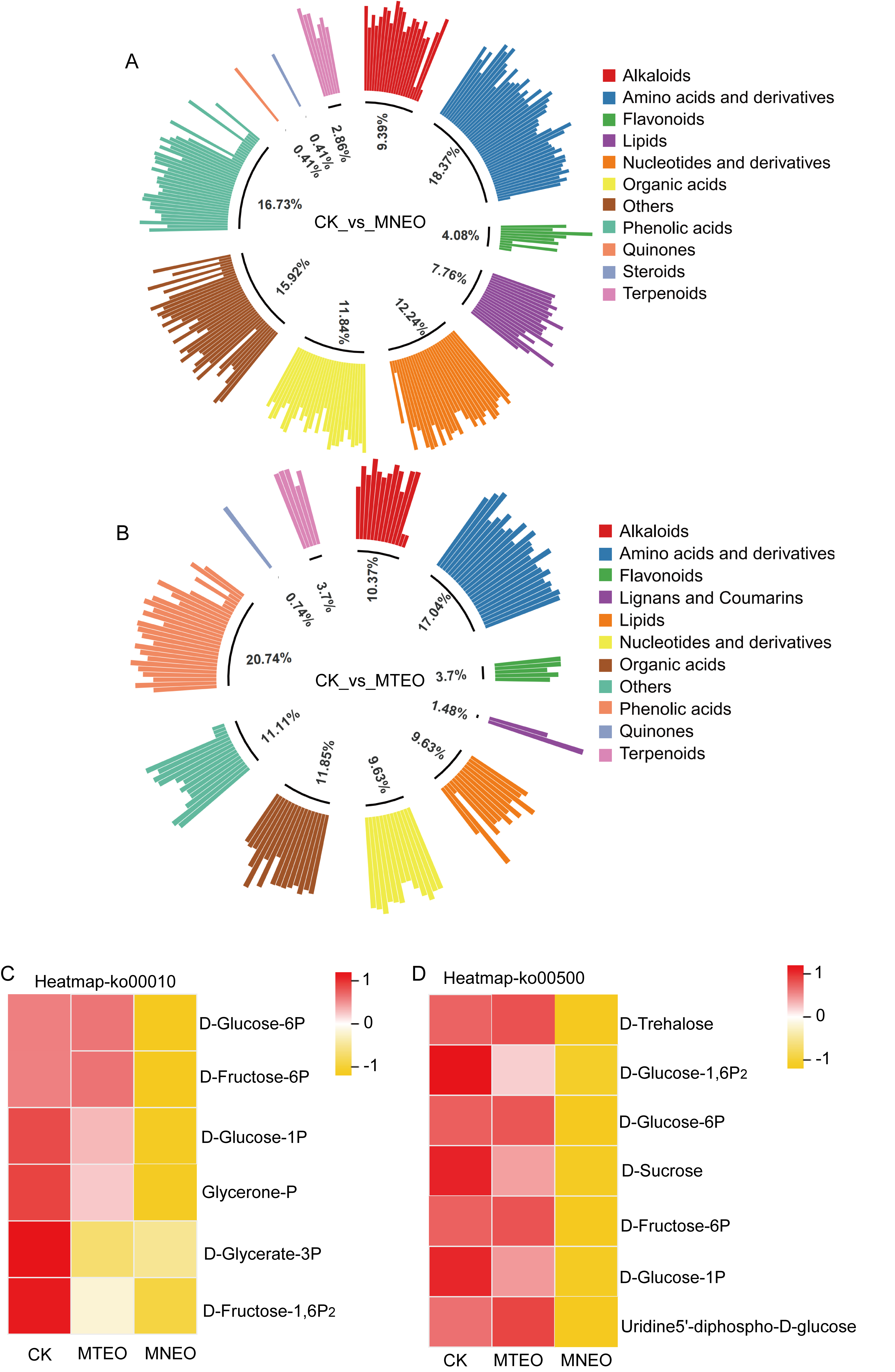


**Figure S3** Metabolite differences were analyzed following treatment of *F. oxysporum* with MNEO and MTEO. (A) MNEO-treated group; (B) MTEO-treated group. (C, D) Heatmaps of saccharide metabolites treated with MNEO and MTEO changed significantly. ko00500 indicates starch and sucrose metabolism, ko00010 indicates glycolysis/gluconeogenesis. D-Glucose-6P: D-glucose-6-phosphate, D-Fructose-6P: D-fructose-6-phosphate, D-Glucose-1P: D-glucose-1-phosphate, Glycerone-P: glycerone phosphate, D-Glycerate-3P: D-glycerate-3-phosphate, D-Fructose-1,6P_2_: D-fructose-1,6-bisphosphate, D-Glucose-1,6P_2_: D-glucose-1,6-bisphosphate, D-Glyceraldehyde-3P: D-glyceraldehyde-3-phosphate.


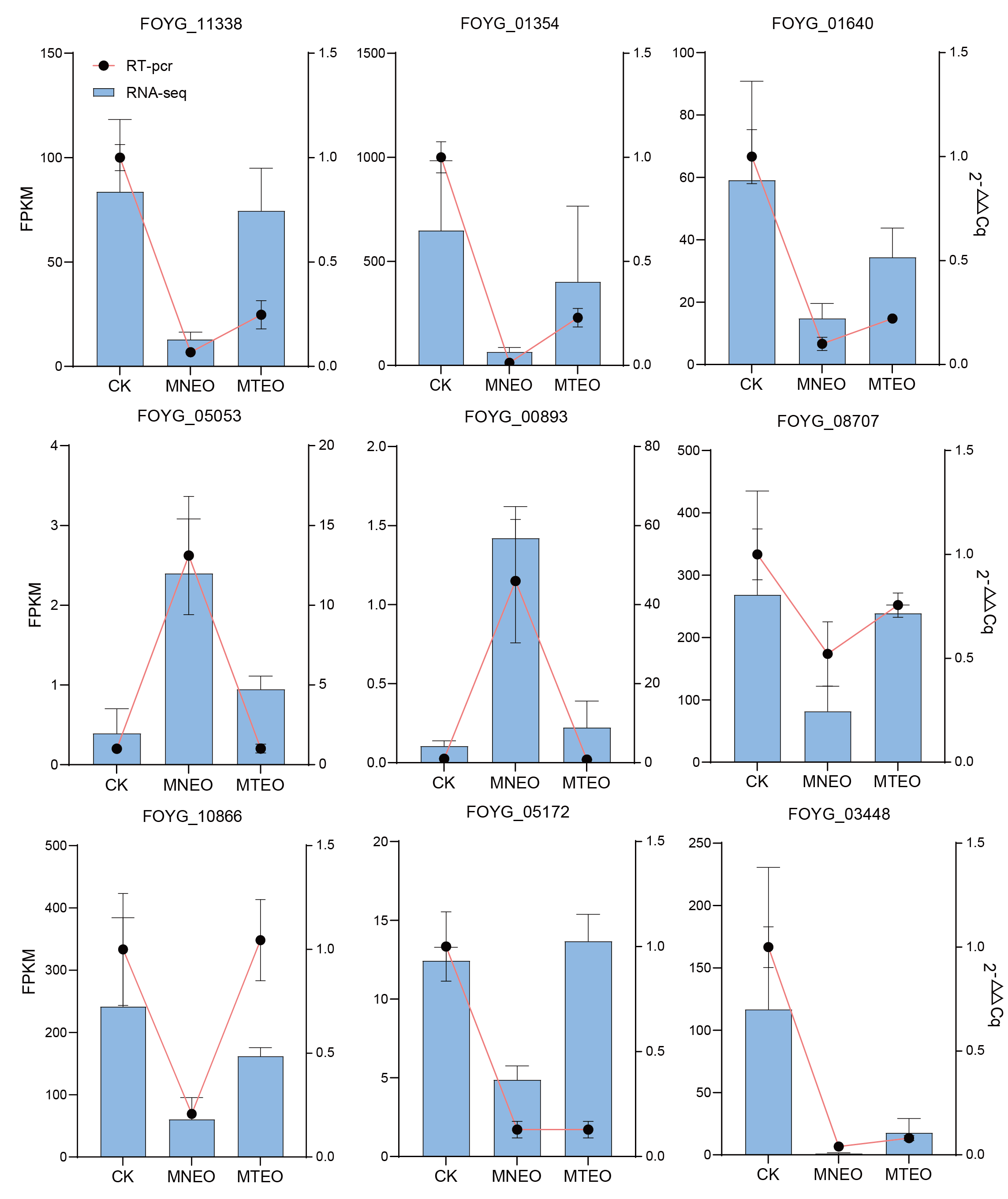


**Figure S4** Effect of MNEO and MTEO on the expression of deacetylase-regulated genes. Each RT-qPCR assay was repeated three times. Bars = mean ± SD from three technical replicates

| **Table S1** DEGs for metabolic pathways enriched in the MNEO and MTEO treatment groups | | | | | | | | |
| --- | --- | --- | --- | --- | --- | --- | --- | --- |
| KEGG pathway | Gene ID | CK_vs_MTEO | | | CK_vs_MNEO | | | KEGG |
|  |  | log2FoldChange | .padj | regulated | log2FoldChange | .padj | regulated |  |
| Meiosis (ko04113) | FOYG_01109 | -7.33 | 0.00 | down | -8.17 | 0.00 | down | K08139 MFS transporter, SP family, sugar:H+ symporter (  HXT) |
|  | FOYG_01912 | -6.27 | 0.00 | down | -5.87 | 0.00 | down |  |
|  | FOYG_03740 | -2.31 | 0.06 | false | -7.14 | 0.00 | down |  |
|  | FOYG_13335 | -1.54 | 0.01 | down | -2.92 | 0.00 | down |  |
|  | FOYG_15653 | -1.81 | 0.04 | down | -1.77 | 0.02 | down |  |
|  | FOYG_00350 | -1.57 | 0.08 | false | -3.25 | 0.00 | down |  |
|  | FOYG_04208 | -1.94 | 0.11 | false | -5.61 | 0.00 | down |  |
|  | FOYG_05514 | -2.51 | 0.01 | down | -3.20 | 0.00 | down |  |
|  | FOYG_05660 | -0.60 | 0.51 | false | -2.40 | 0.02 | down |  |
|  | FOYG_06867 | -0.71 | 0.44 | false | -2.44 | 0.00 | down |  |
|  | FOYG_08813 | -2.56 | 0.00 | down | -3.96 | 0.00 | down |  |
|  | FOYG_11966 | -1.48 | 0.11 | false | -4.03 | 0.00 | down |  |
|  | FOYG_12834 | -1.78 | 0.03 | down | -3.14 | 0.00 | down |  |
|  | FOYG_14383 | -2.14 | 0.01 | down | -3.00 | 0.00 | down |  |
|  | FOYG_14398 | -1.89 | 0.05 | false | -3.75 | 0.00 | down |  |
|  | FOYG_14714 | -0.96 | 0.12 | false | -3.73 | 0.00 | down |  |
|  | FOYG_14790 | -5.37 | 0.00 | down | -5.38 | 0.00 | down |  |
|  | FOYG_14837 | -3.53 | 0.00 | down | -3.32 | 0.00 | down |  |
|  | FOYG_14991 | -1.35 | 0.14 | false | -3.58 | 0.00 | down |  |
|  | FOYG_15416 | -3.18 | 0.00 | down | -5.62 | 0.00 | down |  |
|  | FOYG_05827 | -1.14 | 0.43 | false | -6.96 | 0.00 | down |  |
|  | FOYG_11238 | -1.17 | 0.03 | down | -1.47 | 0.00 | down |  |
|  | FOYG_05827 | -1.14 | 0.43 | false | -6.96 | 0.00 | down | K04630 guanine nucleotide-binding protein G(i) subunit alpha (  GNAI) |
|  | FOYG_11238 | -1.17 | 0.03 | down | -1.47 | 0.00 | down |  |
|  | FOYG_12160 | -1.04 | 0.14 | false | -2.01 | 0.00 | down | K04563 cyclin-dependent kinase (  CDC28) |
|  | FOYG_15599 | 0.39 | 0.60 | false | -3.06 | 0.00 | down |  |
|  | FOYG_06834 | -0.52 | 0.62 | false | -2.02 | 0.02 | down | K06639 cell division cycle 14 (CDC14) |
|  | FOYG_05172 | -0.24 | 0.73 | false | -1.82 | 0.00 | down | K12769 meiosis-specific transcription factor NDT80 (NDT80) |
|  | FOYG_01590 | -1.52 | 0.07 | false | -2.04 | 0.01 | down | K03353 anaphase-promoting complex subunit 6 (APC6) |
|  | FOYG_05721 | -0.48 | 0.57 | false | -1.21 | 0.02 | down | K12761 carbon catabolite-derepressing protein kinase (SNF1) |
|  | FOYG_05087 | -0.36 | 0.61 | false | -1.11 | 0.02 | down | K07827 GTPase KRas (KRAS) |
|  | FOYG_06928 | -0.33 | 0.79 | false | -1.99 | 0.02 | down | K06662 cell cycle checkpoint protein (HRAD17) |
| Glycolysis / Gluconeogenesis (ko00010) | FOYG_01634 | -2.32 | 0.01 | down | -3.33 | 0.00 | down | K15633 2,3-bisphosphoglycerate-indep endent phosphoglycerate mutase (gpmI) |
|  | FOYG_07546 | -4.38 | 0.00 | down | -1.75 | 0.00 | down | K13953 alcohol dehydrogenase, propanol-preferring (adhP) |
|  | FOYG_13381 | -0.80 | 0.48 | false | -5.83 | 0.00 | down |  |
|  | FOYG_10851 | -2.81 | 0.00 | down | -6.51 | 0.00 | down |  |
|  | FOYG_15822 | -5.66 | 0.00 | down | -6.98 | 0.00 | down |  |
|  | FOYG_02647 | -2.31 | 0.04 | down | -3.45 | 0.00 | down | K01803 triosephosphate isomerase (TIM) |
|  | FOYG_07357 | -1.11 | 0.18 | false | -3.44 | 0.00 | down | K01689 enolase 1/2/3 (  ENO1_2_3, eno) |
|  | FOYG_00446 | -3.15 | 0.00 | down | -5.28 | 0.00 | down | K01624 fructose-bisphosphate aldolase, class II (FBA) |
|  | FOYG_08437 | 0.04 | 0.98 | false | -2.91 | 0.00 | down |  |
|  | FOYG_01201 | -2.79 | 0.00 | down | -5.10 | 0.00 | down | K01568 pyruvate decarboxylase (PDC) |
|  | FOYG_13030 | -2.58 | 0.02 | down | -6.27 | 0.00 | down |  |
|  | FOYG_14954 | -5.48 | 0.00 | down | -6.79 | 0.00 | down | K01007 pyruvate, water dikinase (pps) |
|  | FOYG_12714 | -2.12 | 0.02 | down | -3.56 | 0.00 | down | K00927 phosphoglycerate kinase (PGK) |
|  | FOYG_00681 | 1.13 | 0.45 | false | -6.12 | 0.00 | down | K00128 aldehyde dehydrogenase (NAD+) (ALDH) |
|  | FOYG_01972 | -1.71 | 0.05 | false | -4.32 | 0.00 | down |  |
|  | FOYG_09130 | -5.49 | 0.00 | down | -8.70 | 0.00 | down |  |
|  | FOYG_00476 | 1.77 | 0.30 | false | -5.66 | 0.00 | down |  |
|  | FOYG_06370 | -1.46 | 0.17 | false | -4.58 | 0.00 | down |  |
|  | FOYG_00667 | -3.38 | 0.00 | down | -1.84 | 0.01 | down | K00002 alcohol dehydrogenase (NADP+) (AKR1A1) |
|  | FOYG_01905 | -2.23 | 0.01 | down | -0.91 | 0.26 | false | K01810 glucose-6-phosphate isomerase (GPI) |
|  | FOYG_03676 | -1.93 | 0.04 | down | -1.58 | 0.08 | false | K00873 pyruvate kinase (PK) |
|  | FOYG_09920 | 0.79 | 0.12 | false | -1.63 | 0.00 | down | K01610 phosphoenolpyruvate carboxykinase (ATP) (pckA) |
|  | FOYG_06786 | -0.84 | 0.36 | false | -2.23 | 0.00 | down | K00844 hexokinase (HK) |
|  | FOYG_09669 | -0.79 | 0.13 | false | -2.19 | 0.00 | down |  |
|  | FOYG_01354 | -1.12 | 0.29 | false | -3.90 | 0.00 | down | K00134 glyceraldehyde 3-phosphate dehydrogenase (phosphorylating) (GAPDH) |
| Starch and sucrose metabolism(ko00500) | FOYG_09134 | -2.15 | 0.01 | down | -8.59 | 0.00 | down | K05349 beta-glucosidase (bglX) |
|  | FOYG_12849 | -3.01 | 0.03 | down | -5.37 | 0.00 | down |  |
|  | FOYG_13073 | -1.49 | 0.06 | false | -4.90 | 0.00 | down |  |
|  | FOYG_05938 | -2.82 | 0.00 | down | -4.14 | 0.00 | down |  |
|  | FOYG_15281 | -2.76 | 0.03 | down | -2.73 | 0.02 | down |  |
|  | FOYG_07245 | -2.78 | 0.00 | down | -1.55 | 0.02 | down | K01210 glucan 1,3-beta-glucosidase (E3.2.1.58) |
|  | FOYG_01137 | -4.67 | 0.00 | down | -3.95 | 0.00 | down | K01199 glucan endo-1,3-beta-D-glucosidase (E3.2.1.58) |
|  | FOYG_04874 | -2.56 | 0.00 | down | -2.00 | 0.00 | down |  |
|  | FOYG_02005 | -7.87 | 0.00 | down | -7.92 | 0.00 | down | K01182 oligo-1,6-glucosidase (IMA) |
|  | FOYG_07622 | -2.07 | 0.01 | down | -1.62 | 0.01 | down |  |
|  | FOYG_02901 | -3.41 | 0.00 | down | -7.60 | 0.00 | down | K00693 glycogen synthase (GYS) |
|  | FOYG_04825 | -1.75 | 0.03 | down | -2.84 | 0.00 | down | K00688 glycogen phosphorylase (PYG) |
|  | FOYG_05959 | -3.03 | 0.01 | down | -1.27 | 0.10 | false | K18447 ADP-sugar diphosphatase (NUDX14) |
|  | FOYG_01905 | -2.23 | 0.01 | down | -0.91 | 0.26 | false | K01810 glucose-6-phosphate isomerase (GPI) |
|  | FOYG_11338 | -0.62 | 0.44 | false | -3.26 | 0.00 | down | K01194 alpha,alpha-trehalase (TREH) |
|  | FOYG_11832 | -1.85 | 0.06 | false | -3.45 | 0.00 | down | K01193 beta-fructofuranosidase (INV) |
|  | FOYG_09687 | -1.39 | 0.11 | false | -2.97 | 0.00 | down |  |
|  | FOYG_16531 | 0.03 | 0.98 | false | -4.73 | 0.00 | down | K01188 beta-glucosidase (E3.2.1.21) |
|  | FOYG_14356 | 0.25 | 0.86 | false | -4.60 | 0.00 | down | K01179 endoglucanase (E3.2.1.4) |
|  | FOYG_06786 | -0.84 | 0.36 | false | -2.23 | 0.00 | down | K00844 hexokinase (HK) |
|  | FOYG_09669 | -0.79 | 0.13 | false | -2.19 | 0.00 | down |  |
|  | FOYG_00544 | -0.94 | 0.41 | false | -2.12 | 0.00 | down | K00750 glycogenin (GYG1) |
|  | FOYG_01640 | -1.25 | 0.12 | false | -2.58 | 0.00 | down | K00697 trehalose 6-phosphate synthase (otsA) |

| **Table S2** DEGs of metabolic pathways enriched in the MNEO treatment group (Top10) | | | | | |
| --- | --- | --- | --- | --- | --- |
| KEGG pathway | Gene ID | CK_vs_MNEO | | | KEGG |
|  |  | log2FoldChange | .padj | regulated |  |
| ribosome (ko03010) | FOYG_04619 | -1.51 | 0.00 | down | K02959 small subunit ribosomal protein S16 |
|  | FOYG_01340 | -1.55 | 0.03 | down | K02947 small subunit ribosomal protein S10e |
|  | FOYG_01318 | -1.63 | 0.04 | down | K02925 large subunit ribosomal protein L3e |
|  | FOYG_08638 | -1.63 | 0.02 | down | K02966 small subunit ribosomal protein S19e |
|  | FOYG_09765 | -1.77 | 0.02 | down | K02936 large subunit ribosomal protein L7Ae |
|  | FOYG_10731 | -1.89 | 0.00 | down | K02976 small subunit ribosomal protein S26e |
|  | FOYG_08142 | -1.98 | 0.01 | down | K02891 large subunit ribosomal protein L22e |
|  | FOYG_06513 | -1.98 | 0.02 | down | K02877 large subunit ribosomal protein L15e |
|  | FOYG_08707 | -2.00 | 0.00 | down | K02893 large subunit ribosomal protein L23Ae |
|  | FOYG_10866 | -2.29 | 0.00 | down | K02898 large subunit ribosomal protein L26e |
| ribosome biogenesis  in eukaryotes (ko03008) | FOYG_03352 | -1.84 | 0.01 | down | K14539 large subunit GTPase 1 |
|  | FOYG_08713 | -1.97 | 0.00 | down | K14532 ribonuclease MRP protein subunit RMP1 |
|  | FOYG_05069 | -2.04 | 0.01 | down | K14569 ribosome biogenesis protein BMS1 |
|  | FOYG_03447 | -2.05 | 0.03 | down | K14558 periodic tryptophan protein 2 |
|  | FOYG_12450 | -2.15 | 0.00 | down | K03538 ribonuclease P protein subunit POP4 |
|  | FOYG_01379 | -2.19 | 0.00 | down | K03685 ribonuclease III |
|  | FOYG_06658 | -2.62 | 0.01 | down | K13288 oligoribonuclease |
|  | FOYG_11675 | -2.68 | 0.01 | down | K11883 RNA-binding protein NOB1 |
|  | FOYG_14847 | -6.83 | 0.00 | down | K14570 RNA exonuclease |
|  | FOYG_03448 | -7.33 | 0.00 | down | K14558 periodic tryptophan protein 2 |
| The above DEGs were all top 10 of each pathway. | | | | | |

| **Table S3** RT-qPCR primer sequences | | |
| --- | --- | --- |
| Gene | L | R |
| FOYG_00893 | CTGAGAATGGCACGTGGAGT | TGAGTGTCAACGGCTCTGTG |
| FOYG_05053 | CGACTTTACTCAAACCATGGCAA | TTGTTTTCGCTTCGCTTCCG |
| FOYG_01354 | GAGCCTCACTACGCCGTCTA | GGTCGCGCTCAGAGTAGAAC |
| FOYG_11338 | GGTCGTTTCCGTGAGCCTTA | GGAGGCTGAGATCGGTTGAG |
| FOYG_01640 | GGCCGTTTTGTGACAGTTGG | AGAGCGTGCAGTTTCTGAGG |
| FOYG_05172 | ACTACGAATCTTCGACGCCC | GGACTGGCGGATGGAATAGG |
| FOYG_03448 | TCCCCTCATGTTGGCGAGAT | ACTCTGGCCAAGCTTCATCC |
| FOYG_10866 | CCTCCGTGTACCGTCTCAAG | GGTGACCTTGTTGTTGGCAG |
| FOYG_08707 | TGCTTTCAATAACAGTTCAGCC | CGAGGTGATCAGGGTCTTGG |
| QTUB | TTCTGCTGTCATGTCCGGTGT | TCAGAGGAGCAAAGCCAACCA |
